# Langqing Meiduo Jiujie pills treatment attenuates acute liver injury in animals by regulating anti-oxidative stress and liver metabolism

**DOI:** 10.3389/fphar.2025.1582435

**Published:** 2025-07-22

**Authors:** Zhongyuan Wang, Fangjie Wang, Yan Huang, Gebai Zhaxi, Jiaqing Fu, Guili Song, Bai Bai, Mengtian Han, Jingwen Zhang, Yiye Li, Ran Li, Ting Zhang, Tsedien Nhamdriel, Yongzhong Zeweng, Chenlei Ganghuan, Zhang Wang

**Affiliations:** ^1^ College of Pharmacy, Chengdu University of Traditional Chinese Medicine, Chengdu, China; ^2^ State Key Laboratory of Southwestern Chinese Medicine Resources, Chengdu University of Traditional Chinese Medicine, Chengdu, China; ^3^ College of Ethnomedicine, Chengdu University of Traditional Chinese Medicine, Chengdu, China; ^4^ The department of Tibetan medicine, Xizang University of Tibetan Medicine, Lhasa, China; ^5^ National Inheritance Studio for Famous Doctor of Traditional Chinese Medicine (Tibetan Doctor: Jiangyong Silang), Chengdu University of Traditional Chinese Medicine, Chengdu, China

**Keywords:** Langqing Meiduo Jiujie pills, dose-effect relationship, acute drug-induced liver injury, chemical acute liver injury, liver metabolomics

## Abstract

**Introduction:**

Langqing Meiduo Jiujie pills (LQMDJJP), a Tibetan formula, has a history of more than 400 years of clinical use. However, there has been no report on the scientific basis of its dosage or its mechanism of action in treating acute liver injury. To investigate the optimal clinical dosage of LQMDJJP, to examine the differences in differential metabolites in liver tissue following treatment with LQMDJJP, and to explore the mechanism through which LQMDJJP acts in the treatment of acute liver injury.

**Materials and Methods:**

HE staining was used to observe the pathological changes of the liver, and the Suzuki pathological score was counted. The levels of ALT (Alanine aminotransferase), AST (Aspartate aminotransferase), TBIL (Total bilirubin), DBIL (Direct bilirubin), SOD (Superoxide dismutase), MDA (Malondialdehyde), GSH (Glutathione) and GSSG (Glutathione disulfide) and were detected by colorimetry kit. UPLC-Q-TOF-MS metabolomics technology was used to explore the differential metabolites and differential metabolic pathways of LQMDJJP in the treatment of acute liver injury. PCR and WB were employed to confirm the mechanism of LQMDJJP in treating acute liver injury via the Keap1-Nrf2 to anti-oxidative stress pathway.

**Results:**

This study found that the optimal dose of LQMDJJP in the treatment of C57BL/6 mice was 333.33 mg/kg/d, and the optimal dose of LQMDJJP in the treatment of SD rats was 166.66 mg/kg/d. It was found that LQMDJJP can improve the morphological state and pathological changes of the liver, significantly reduce the levels of ALT, AST, TBIL, DBIL, SOD and GSH, and also increase the levels of MDA and GSSG. UPLC-Q-TOF-MS metabolomics technology screened 121 metabolic differences and six metabolic pathways that met the screening conditions. It was found that the treatment of acute liver injury by LQMDJJP may be related to the metabolism of glutamic acid, glutamine and γ-glutamylalanine. LQMDJJP can reduce the gene and protein expression levels of Keap1 and can increase the gene and protein expression levels of Nrf2, HO1, NQO1, GCLC and other oxidative stress indicators.

**Discussion:**

LQMDJJP can significantly improve acute liver injury induced by CCl_4_ and APAP, and the clinical dosage is reasonable, and its protective effect against APAP-induced acute liver injury is mediated through the Keap1-Nrf2 to anti-oxidative stress mechanism.

## Graphical Abstract

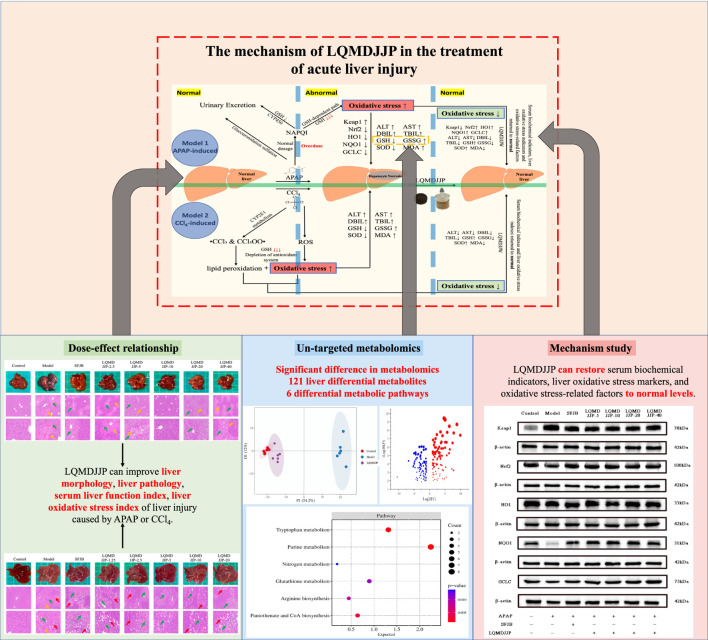

## 1 Introduction

In contemporary society, liver injury has become an increasingly pressing health issue. The liver, being the body’s largest and most vital metabolic organ, plays a crucial role in various physiological functions, including detoxification, metabolism, and the synthesis of essential proteins (Tanwar et al., 2020; Xue and Chen, 2022). Based on the varying etiological factors, liver injury can be classified into drug-induced liver injury, chemical-induced liver injury, alcoholic liver injury, cholestatic liver injury, and others (Deng and Ma, 2022). Drug-induced liver injury refers to liver injury by the excessive use of medications. This may occur when the active metabolites of the drugs in the body surpass the maximum threshold the body can manage, leading to a series of reactions such as oxidative stress and inflammation (Yu et al., 2019). Chemical-induced liver injury results from exposure to or ingestion of various toxic substances, including industrial chemicals, environmental pollutants, and certain plant toxins. It typically involves direct cytotoxic effects, which can cause direct damage or necrosis of hepatocytes, such as those caused by carbon tetrachloride, nitrites, and others (Tan et al., 2022).

Acetaminophen (APAP), a commonly used medication for treating colds, is among the most frequent drugs associated with drug-induced liver injury (Ramachandran and Jaeschke, 2018). Research has indicated that oxidative stress, inflammation, mitochondrial dysfunction, endoplasmic reticulum stress (ERS), and disruption of Ca2^+^ homeostasis are linked to the pathogenesis of APAP-induced liver injury (AILI) (Kaplowitz, 2005; Yan et al., 2018). At therapeutic doses, the acetaminophen metabolite NAPQI is typically neutralized by binding to glutathione, forming a non-toxic compound that is then excreted in the urine (Graham et al., 2005; Hodgman and Garrard, 2012). However, when the recommended dose is surpassed, the liver’s metabolic capacity may become overwhelmed, depleting glutathione reserves. As a result, NAPQI cannot be adequately neutralized, leading to its binding with proteins and lipids in hepatocytes, causing oxidative stress, inflammation, cellular damage, and necrosis (Moles et al., 2018; Tirmenstein and Nelson, 1989).

Carbon tetrachloride (CCl_4_), a widely used chemical reagent, is also a potent hepatotoxic agent. Accidental ingestion, as well as eye and skin exposure, can result in severe chemical-induced liver damage (Chen and Qi, 2011). CCl_4_ is metabolized in the liver, where cytochrome P_450_ enzymes convert it into active metabolites, including chloroform and trichloromethyl peroxyl radical (Chen et al., 2000). These metabolites are highly toxic and can harm liver cells. The active metabolites can trigger lipid peroxidation, resulting in damage to the cell membranes (Ueno and Komatsu, 2017; Zhou and Deng, 2018). This damage can compromise the integrity and function of cells, ultimately causing cell death. The metabolism of CCl_4_ generates a significant number of free radicals, which results in heightened oxidative stress (Xiao et al., 2019). Free radicals can harm proteins, lipids, and DNA within cells, exacerbating liver injury. Damaged hepatocytes release inflammatory mediators, initiating both local and systemic inflammatory responses, which contribute to additional liver damage. Prolonged exposure to CCl_4_ can result in liver fibrosis and cirrhosis, driven by ongoing hepatocyte injury and chronic inflammation (Feng et al., 2011; Gong, 2019).

Under the influence of drug metabolism or external stimuli, reactive oxygen species (ROS) free radicals are generated (Forbes et al., 2008). The liver possesses various antioxidant systems, including glutathione, superoxide dismutase, and catalase, which typically neutralize and remove free radicals under normal conditions. However, when excessive free radicals are produced or the antioxidant defense mechanisms become compromised, oxidative stress is triggered, leading to lipid peroxidation and the oxidation of macromolecules (Dröge, 2002). Oxidative stress is a key factor in the progression of various chronic liver diseases, including hepatitis, nonalcoholic fatty liver disease, liver fibrosis, and cirrhosis (Zhao et al., 2019). When oxidative stress arises, the interaction between Keap1 and Nrf2 is inhibited. Free Nrf2 is translocated from the cytoplasm to the nucleus, where it binds to the antioxidant response element (ARE) to regulate the activities of downstream enzymes like HO-1, NQO1, GCLC, SOD, CAT, and GSH-Px, aiding in the elimination of harmful substances such as ROS (Chen et al., 2023; Ding, 2023). Oxidative stress can also trigger additional signaling pathways, such as the NF-kB and JNK pathways, which promote inflammation and apoptosis. Injured cells release inflammatory mediators, initiating both local and systemic inflammatory responses, thereby worsening liver injury. The activation of the p62-keap1-Nrf2 antioxidant pathway may have a protective effect in APAP-induced acute liver injury (Shen et al., 2018). Nrf2 activators may enhance Nrf2-ARE signaling by modulating cell proliferation, apoptosis, and antioxidant defense mechanisms, thereby counteracting hepatotoxicity (Jayasuriya et al., 2021). Lu et al. discovered that the active compounds in *Veronica ciliata* Fisch. play a protective effect on the liver by activating the p62-Keap1-Nrf2 pathway (Lu et al., 2019). Carolina I. Ghanem et al. discovered that Nrf2 regulates the transcription of ABC transporters in the brain during APAP-induced acute toxicity in mice (Ghanem et al., 2015). Recent pharmacological research has revealed that scutellarin can protect against acute alcoholic liver injury by modulating the Nrf2/HO-1 pathway to counteract oxidative stress (Zhang et al., 2023).

Metabolomics investigates biological systems by analyzing the alterations in metabolites or their variations over time following the stimulation or disruption of biological systems like cells and tissues (Ma et al., 2022). Xingbo Bian et al. applied metabolomics techniques to discover that the Tibetan medicine Qiwei Tiexie pills can ameliorate metabolic disturbances induced by APAP, thereby reducing inflammation and oxidative stress resulting from an APAP overdose (Bian et al., 2024). Shouer Lin et al. investigated the treatment of alcoholic liver disease with Pien-Tze-Huang using metabolomics technology and identified 80 differentially expressed metabolites. They also found that PTH exerts a hepatoprotective effect by regulating the inflammatory cytokine signaling pathway, primary bile acid biosynthesis, vitamin B6 metabolism, cholesterol metabolism, and tyrosine metabolism (Lin et al., 2023). Zhichao Liu et al. employed 1H-NMR metabolomics to assess the risk of acute hepatotoxicity and nephrotoxicity associated with Ershiwuwei Songshi pills (Liu et al., 2021).

LQMDJJP was initially documented in the Tibetan medicine classic *Tibetan Medicine Recipe Supplement*, compiled by the renowned Tibetan medicine scholar Dist Sangjiejiacuo in the 17th century (Gyatso, 1991). It is also recorded in *Drug Standards of the People‘s Republic of China: Tibetan Medicine* (Health, 1998), *National Tibetan Medicine Standard Book 3* (Wang, 2004), *Data Mining and Application of Classic Prescriptions of Chinese Ethnic Medicine* (Xie and Li, 2024) and *Modern Research and Clinical Application of Tibetan Medicine Formulas* (Zhan and Zhao, 2009). It has a history of more than 400 years of clinical use. It is composed of *Bovis Calculus* Artifactus*, Pterocarpus indicus* Willd.*, Aquilaria sinensis* (Lour.) Spreng.*, Phyllanthus emblica* L.*, Meconopsis* spp. and other medicinal materials (Table 1). It has the properties of clearing heat and detoxifying, soothing the liver, and regulating qi. Clinically, it is used to treat conditions such as ‘Qinchamubu’ disease, hepatitis, fatty liver, alcoholic liver disease, liver cirrhosis with symptoms like liver pain, abdominal distension, abdominal pain, chest and back pain, as well as ‘Gangban’ disease, which manifests with symptoms such as dizziness, headache, chest and back pain, chest tightness, shortness of breath, lip cyanosis, and gingival bleeding.

**TABLE 1:** The drugs contained in LPMDJJP and the proportion of each drug in the preparation.

No.	Ingredients	Proportion (%)
1	*Bovis Calculus* Artifactus	12.94
2	*Santalum album* L.	3.56
3	*Pterocarpus indicus* Willd.	2.59
4	*Aquilaria sinensis* (Lour.) Spreng.	0.65
5	*Saxifraga bulleyana* Engl. and Irmsch.	1.62
6	*Corydalis hendersonii* Hemsl.	6.47
7	*Phyllanthus emblica* L.	5.50
8	*Meconopsis* spp.	3.24
9	*Herpetospermum pedunculosum* (Ser.) C. B. Clarke.	0.32
10	*Inula racemosa* Hook.f.	3.24
11	*Coriandrum sativum* L.	5.83
12	*Dracocephalum tanguticum* Maxim.	3.24
13	*Faeces Trogopterpri* Extract	11.97
14	*Aucklandia lappa* Decne.	6.47
15	Concretio Silicea Bambusae	9.39
16	*Carthamus tinctorius* L.	11.33
17	*Crocus sativus* L.	4.85
18	*Corydalis racemosa* (Thunb.) Pers.	5.18
19	*Imperata cylindrica* (L.) Beauv.	1.62

**FIGURE 1:**
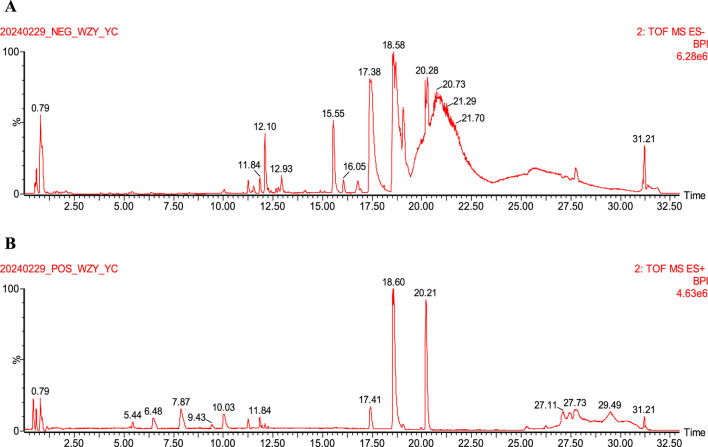
UPLC-Q-TOF-MS fingerprint of LQMDJJP. **(A)** is an isogram in negative ion mode. **(B)** is the spectrum in positive ion mode.

The LQMDJJP samples were analyzed using Waters ACQUITY UPLC CHS C18 column (150 mm × 2.1 mm, 1.7 μm) at a temperature of 35°C. The mobile phase A consisted of 95% water and 5% acetonitrile (with 0.1% formic acid), while mobile phase B was acetonitrile. The gradient elution program was as follows: 0–3.5 min (1.5%–20% B), 3.5–19 min (20%–50% B), 19–26 min (50%–95% B), 26–30 min (95%–1.5% B), 30–33 min (1.5% B). The flow rate was set at 0.2 mL/min, and the sample injection volume was 2 μL. Electrospray ionization ion source (Waters, United States) and mass spectrometry module (Waters, United States) were used to detect LQMDJJP samples. The ion source temperature was 120°C, the desolvation temperature was 400°C, the desolvation flow rate was 1000 L/hr, the capillary voltage was 2.5 KV, the cone hole voltage was 25 V, the scanning area was 100∼1,200 amu, the positive ion and negative ion modes were tested respectively, and the mass charge ratio (m/z) range was 100∼1,200 Da full scan. The UPLC-Q-TOF fingerprint of LQMDJJP is shown in Figure 1.

This study aims to replicate the most common drug-induced and chemical-induced acute liver injury models using CCl_4_ and APAP, employing LC-MS metabolomics and molecular biology techniques such as PCR and WB. The therapeutic efficacy of LQMDJJP in treating acute liver injury was assessed, the dose-response relationship was investigated, and the mechanism of LQMDJJP’s action in alleviating acute liver injury through anti-oxidative stress was explored, providing a foundation for its clinical application.

## 2 Materials and methods

### 2.1 Drugs, reagents and antibodies

LQMDJJP were sourced from Xizang Xiongbalaqu Shenshui Tibetan Medicine Co., Ltd., with the record No. Z20211255000 and the batch No. 20220401. Silibinin capsules were sourced from Tianjin Taslyst Pharmaceutical Co., Ltd., with approval No. H20040299 and the batch No. 350710071. Pian Zai Huang was obtained from Zhangzhou Pien Tze Huang Pharmaceutical Co., Ltd., with approval No. Z35020243 and the batch No.2311189.

Acetaminophen was obtained from Jingpu Xitang Biotechnology Co., Ltd., with the batch No. C22PA8858. Carbon tetrachloride and olive oil were sourced from Shanghai Yien Chemical Technology Co., Ltd. Uratan was purchased from Chengdu Cologne Chemical Co., Ltd., with the batch No. 2022032101.

β-actin (P60710) was purchased from Wuhan Servicebio Technology Co., Ltd.; Keap1 Polyclonal antibody (10503-2-AP), NFE2L2 Recombinant antibody (80593-1-RR), NQO1 Polyclonal antibody (11451-1-AP), HO-1/HMOX1 Polyclonal antibody (10701-1-AP), GCLC Polyclonal antibody (12601-1-AP) and Multi-rAb HPR-Goat Anti-Rabbit Recombinant Secondary Antibody (RGAR001) were purchased from Proteintech Group, Inc.

### 2.2 Animals

Eighty-two male SD rats, SPF grade, weighing between 180 and 220 g, and ninety-four male C57BL/6 mice, SPF grade, weighing between 18 and 22 g, were sourced from Chengdu Dashuo Experimental Animal Co., Ltd. The laboratory animal production license number is SCXK (Sichuan) 2020-0030, and the laboratory animal quality certificate numbers are No. 51203500046625 and No. 51203500047155.

This research was carried out following the guidelines set by the Ministry of Science and Technology’s *Guidelines for the Treatment and Use of Laboratory Animals*. All animal experiments received approval from the Animal Care and Use Committee of Chengdu University of Traditional Chinese Medicine and adhered to *the Laboratory Animal Care and Use Guidelines* published by the National Academy of Sciences Press. The ethical review number for animal welfare is 2024063, with the review conducted on 24 April 2024.

### 2.3 Grouping and administration

According to the *Pharmacological Experimental Methodology* (4th Edition) and *Research Methodology in Traditional Chinese Medicine Pharmacology*, the equivalent dose ratio of mice to humans is about 8–12 times (conversion coefficient 0.081–0.135), and the equivalent dose ratio of rats to humans is about 4–6 times (conversion coefficient 0.162–0.243) (Chen et al., 2011; Wei et al., 2010). In this study, based on this principle, we set the equivalent dose of mice to 10 times that of humans, and the equivalent dose of rats to 5 times that of humans. The 10-fold dose set by mice and the 5-fold dose set by rats in the original text are the medium dose of LQMDJJP for the treatment of acute liver injury, which is equivalent to the daily dose of adults. On the basis of the equivalent dose (medium dose), we increased two doses in an equal proportion, as high dose and ultra-high dose. Two doses were reduced downward as low and ultra-low doses. We studied these five LQMDJJP dose groups.

Group setting: The recommended daily dose of LQMDJJP for an adult (average weight 60.00 kg) is 2.00 g/60 kg/d, equivalent to 33.33 mg/kg/d. In this study, the SD rat dose is set to five times that of humans, so the medium dose equivalent for SD rats is 5 times the human daily dose, which equals 166.65 mg/kg/d. For C57BL/6 mice, the equivalent dose is set to ten times that of humans, meaning the equivalent dose for C57BL/6 mice is 10 times the human dose, or 333.33 mg/kg/d.

In the APAP-induced acute liver injury model, C57BL/6 mice were grouped based on body weight and randomly assigned to eight groups: the control group, the model group, the SFJB group (70.00 mg/kg/d), the LQMDJJP-2.5 group (83.33 mg/kg/d), the LQMDJJP-5 group (166.66 mg/kg/d), the LQMDJJP-10 group (333.33 mg/kg/d), the LQMDJJP-20 group (666.66 mg/kg/d) and the LQMDJJP-40 group (1,333.32 mg/kg/d). For the CCl_4_-induced acute liver injury model, SD rats were also stratified by body weight and randomly assigned to eight groups: the control group, the model group, the SFJB group (70.00 mg/kg/d), the LQMDJJP-1.25 group (41.66 mg/kg/d), the LQMDJJP-2.5 group (83.33 mg/kg/d), the LQMDJJP-5 group (166.66 mg/kg/d), the LQMDJJP-10 group (333.33 mg/kg/d) and the LQMDJJP-20 group (666.66 mg/kg/d).

Following 1 week of acclimation for the experimental animals, preventive intragastric administration began, with the treatment lasting for 7 days.

### 2.4 Model replication

Reproduction of the APAP-induced acute liver injury model: APAP powder was carefully weighed, dissolved in normal saline, and vortexed using a vortex mixer until the acetaminophen was fully dissolved. On the seventh day, APAP modeling was started 12 h after prophylactic administration. During the modeling process, the control group received an equivalent volume of normal saline, while the other groups were intraperitoneally injected with APAP at a dosage of 300 mg/kg (An et al., 2023; Li et al., 2023).

Reproduction of the CCl_4_-induced acute liver injury model: CCl_4_ and olive oil were precisely measured and thoroughly mixed. On the seventh day, modeling started 2 h after the preventive administration. During the modeling process, animals in the control group were injected with 2 mL/kg of olive oil, while animals in the other groups received an intraperitoneal injection of the same volume of 40% CCl_4_ olive oil solution for induction (Ni et al., 2024; Xiyu et al., 2023).

### 2.5 Liver morphology and index

12 h after intraperitoneal injection of APAP, or 24 h after intraperitoneal injection of CCl_4_, the animals were anesthetized with a 20% urethane solution at a dosage of 1.2 g/kg body weight. Blood was collected from the abdominal aorta, and the animals were euthanized by cervical dislocation. The liver was then excised, and its morphology was examined. The liver weight was recorded, and the organ index was calculated based on body weight. Liver index = (liver weight/body weight) ×100%.

### 2.6 Pathological analysis

After euthanizing the animals, the liver tissue was carefully removed and rinsed with normal saline to eliminate blood residues. Tissues from the same region were collected and fixed in 4% paraformaldehyde. Following dehydration, the tissues were embedded in paraffin and sliced. The sections were then stained with hematoxylin for nuclear staining, turning the nuclei blue-purple. After washing, the sections were stained with eosin to color the cytoplasm pink. After a final wash, dehydration and transparency treatments were applied to restore the clarity of the slices. Lastly, the sections were sealed with mounting medium, and the results were examined under a microscope and scored based on the scoring principle (Suzuki et al., 1993). The details of the Suzuki score are as follows. The score is based on the degree of sinusoidal congestion in hepatocytes, vacuolar degeneration of hepatocytes, and hepatocyte necrosis. Each parameter is scored on a scale from 0 (none) to 4 (severe), with scores assigned according to the degree of manifestation. The final Suzuki score is the sum of the individual parameter scores, used to quantify the overall severity of liver damage.

### 2.7 Serum biochemical indexes

After euthanizing the animals, whole blood was collected and allowed to stand at room temperature for 30 min. The blood was then frozen and centrifuged at 4°C for 10 min at 3,000 rpm (Rotor diameter 10 cm). The serum was separated and stored at −80°C for subsequent analysis of ALT, AST, TBIL, and DBIL levels.

### 2.8 Liver oxidative stress index

After euthanizing the animals, the liver tissue was stored in a −80°C freezer. One gram of liver tissue was weighed and mixed with 9 mL of normal saline, along with two homogenization beads. The liver tissue was then homogenized using a high-throughput tissue homogenizer at 1,000 rpm (Rotor diameter 10 cm) for three cycles, each lasting 20 s. The fully homogenized liver sample was centrifuged at 3,500 rpm (Rotor diameter 10 cm) and 4°C for 10 min. The upper yellow-clear supernatant was collected as a 10% liver homogenate, which was aliquoted into sterile EP tubes and stored at −20°C. Before biochemical analysis, the samples were thawed on ice, diluted, and their protein concentration was measured using a BCA kit. The levels of MDA, SOD, GSH, and GSSG in the liver tissue were then assessed.

### 2.9 Metabolomics

#### 2.9.1 Sample preparation

12 h after intraperitoneal injection of APAP, the liver tissue was harvested, quickly frozen in liquid nitrogen, and stored at −80°C. For sample preparation, 400 μL of distilled water was added to every 100 mg of tissue, and 200 μL of the homogenate was transferred to a 2.0 mL EP tube. Then, 1,200 μL of acetonitrile precipitant was added. The mixture was vortexed for 2 min to ensure thorough mixing, then centrifuged at 11,000 rpm (Rotor diameter 10 cm) and 4°C for 10 min. The supernatant was collected into a 2 mL EP tube and dried at 37°C using a nitrogen evaporator. It was then re-dissolved in 200 μL of methanol, subjected to ultrasonic treatment for 10 min, vortexed for 2 min, and thoroughly mixed. The sample was then centrifuged at 11,000 rpm (Rotor diameter 10 cm) and 4°C for 10 min. Finally, the supernatant was filtered through a 0.22 μm filter, and the prepared sample was obtained.

#### 2.9.2 Chromatographic conditions

The LQMDJJP samples were analyzed using a Waters ACQUITY UPLC HSS T3 column (100 mm × 2.1 mm i.d., 1.8 μm) at a temperature of 40°C. The mobile phase A consisted of 95% water and 5% acetonitrile (with 0.1% formic acid), while mobile phase B was composed of 47.5% acetonitrile, 47.5% isopropanol, and 5% water (also containing 0.1% formic acid). The gradient elution program was as follows: 0–3 min (0%–20% B), 3–4.5 min (20%–35% B), 4.5–5 min (35%–100% B), 5–6.3 min (100% B), 6.3–6.4 min (100%–0% B), 6.4–8 min (0% B). The flow rate was set at 0.4 mL/min, and the sample injection volume was 3 μL.

#### 2.9.3 Mass spectrometry conditions

The mass spectrometry parameters were as follows: capillary voltage: 3.5 kV (ESI +/−), capillary temperature: 325°C, heating temperature: 425°C, sheath gas flow rate: 50 arb, auxiliary gas flow rate: 13 arb, and scan range: 70–1,050 Da. The scanning modes used were full MS (resolution of 60,000) and MS2 spectra (resolution of 7,500).

#### 2.9.4 Analysis condition

Progenesis QI (Waters Corporation, Milford, United States) software was employed for the analysis of LQMDJJP samples. The MS and MS/MS data were compared with the metabolic database. The MS mass deviation was configured to be under 10 ppm, and metabolite identification was based on the matching score from the secondary mass spectrometry data. PCA and OPLS-DA were conducted on liver tissue sample data. Differential metabolites from liver tissue were identified, meeting the criteria of *P* < 0.05, FC ≥ 2 or ≤ 0.5, and VIP > 1. Functional pathway enrichment using KEGG, topological analysis, and visual representation of the identified metabolites were performed using Metabo Analyst 6.0.

### 2.10 RT-PCR

The reagents and RW1, RW2, RL1 and RL2 buffers in the total RNA extraction kit were purchased from Chengdu Fuji Biotechnology Co., Ltd. Approximately 20 mg of liver tissue was collected, and 500 µL of Buffer RL1 was added before grinding using a liver-spleen model. The resulting mixture was transferred to a DNA removal tube and centrifuged for 2 min to collect the lower liquid. The lower liquid was then combined with 800 µL of Buffer RL2. The resulting solution was transferred into an RNA extraction tube, centrifuged for 1 min, and the lower liquid discarded. Next, 500 µL of Buffer RW1 was added to the RNA extraction tube, centrifuged for 1 min, and the lower liquid removed. 700 μL of Buffer RW2 was added, followed by another 1-min centrifugation and removal of the lower liquid. The tube was centrifuged without any buffer for 1 min. To extract total RNA, 60 µL of RNase-Free ddH2O was added to the RNA extraction tube and centrifuged for 1 min. The reverse transcription reaction was set up according to the instructions, with a 20-min incubation at 42°C, followed by a 5-min inactivation at 85°C. The RNA quantification system was prepared as per the instructions, and PCR detection of the relative gene expression was performed. The primer design is shown in Table 2.

**TABLE 2:** The sequences of the quantitative real-time PCR primers.

Gene	Forward	Reverse
β-actin	CCA​CCA​TGT​ACC​CAG​GCA​TT	CAG​CTC​AGT​AAC​AGT​CCG​CC
Keap1	TGC​CCC​TGT​GGT​CAA​AGT​G	GGT​TCG​GTT​ACC​GTC​CTG​C
Nrf2	TCT​TGG​AGT​AAG​TCG​AGA​AGT​GT	GTT​GAA​ACT​GAG​CGA​AAA​AGG​C
HO1	AAG​CCG​AGA​ATG​CTG​AGT​TCA	GCC​GTG​TAG​ATA​TGG​TAC​AAG​GA
NQO1	AGG​ATG​GGA​GGT​ACT​CGA​ATC	AGG​CGT​CCT​TCC​TTA​TAT​GCT​A
GCLC	GGG​GTG​ACG​AGG​TGG​AGT​A	GTT​GGG​GTT​TGT​CCT​CTC​CC

### 2.11 Western blotting

Approximately 50 mg of liver tissue was homogenized in a liver-spleen mode at 4°C using a homogenizer. The homogenate was then transferred to a 1.5 mL centrifuge tube, frozen, and centrifuged at 12,000 rpm (Rotor diameter 10 cm) and 4°C for 15 min. The supernatant was collected to obtain the total protein. Following the instructions of the BCA protein quantification kit, a standard curve was constructed, and the protein concentration of the sample was calculated. The sample concentrations were adjusted to be uniform by diluting with lysate, and a suitable amount of 5×protein loading buffer was added. The samples were denatured by heating in a metal bath at 100°C for 5–10 min. The processed samples were then stored at −80°C. During the experiment, Western blotting (WB) was performed following standard protocol, including electrophoresis, membrane transfer, blocking, primary antibody incubation, secondary antibody incubation, development, and quantification.

### 2.12 Statistical analysis

The data analysis in this study was conducted using SPSS 22.0 software. One-way ANOVA was employed to assess differences between groups. The *P*-value of less than 0.05 was considered statistically significant.

## 3 Results

### 3.1 Animal mortality and weight changes

**FIGURE 2:**
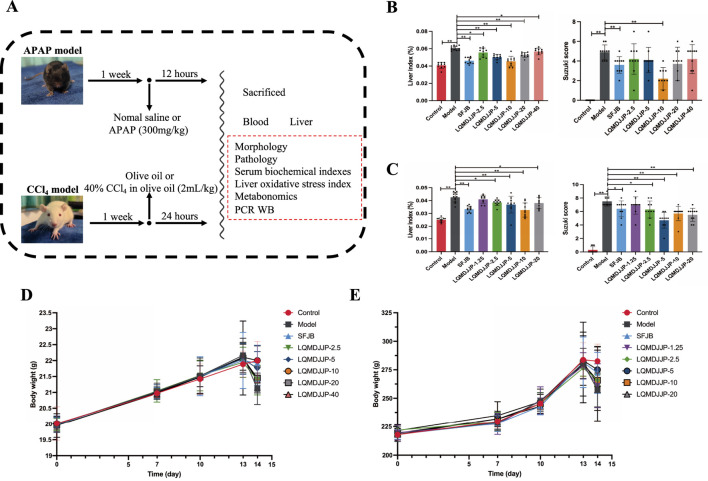
Experimental design, liver index, pathological score and weight change. **(A)** The experimental design of the experiment. **(B)** The liver index and pathological score of mice in APAP-induced acute liver injury model. **(C)** The liver index and pathological score of rats in CCl_4_-induced acute liver injury model. **(D)** The weight change of C57BL/6 mice. **(E)** The weight change of SD rats. Only the model group had a sample size of 12, while the other groups had a sample size of 10. Compared with the model group, *means *P* < 0.05, **means *P* < 0.01.

The experimental procedure is illustrated in Figure 2A. The variations in body weight of animals in the APAP-induced acute liver injury model in mice and the CCl_4_-induced acute liver injury model in rats are presented in Figures 2D,E.

In the APAP-induced acute liver injury model using C57BL/6 mice, no fatalities were observed in any of the groups during the experiment. However, except for the control group, the mice in the other groups exhibited reduced activity, loss of appetite, and a decrease in body temperature within 30 min to 1 h after APAP administration. The model group showed the most pronounced symptoms, which was consistent with the observations from the CCl_4_-induced liver injury model. On the following day, the C57BL/6 mice in the SFJB and LQMDJJP-10 groups showed partial recovery. The body weight of mice in all groups naturally increased over time. The SFJB group, LQMDJJP-5 group, and LQMDJJP-10 group showed notable improvement in preventing weight loss caused by APAP administration, with the LQMDJJP-10 group demonstrating the most significant effect on reversing the weight loss.

In the CCl_4_-induced acute liver injury model, SD rats in all groups survived throughout the experiment. However, except for the control group, the SD rats in the other groups exhibited reduced activity, loss of appetite, and lowered body temperature within 1 h following CCl_4_ intraperitoneal injection, with the model group showing the most pronounced symptoms. On the following day, SD rats in the LQMDJJP-5 and LQMDJJP-10 groups showed partial recovery. The body weight of SD rats in all groups naturally increased over time. Both the SFJB group and the LQMDJJP-5 group demonstrated significant effects in preventing weight loss caused by CCl_4_ administration, with the LQMDJJP-5 group showing the most notable improvement in reversing the weight loss from CCl_4_ intraperitoneal injection.

### 3.2 Liver morphology and organ index

**FIGURE 3:**
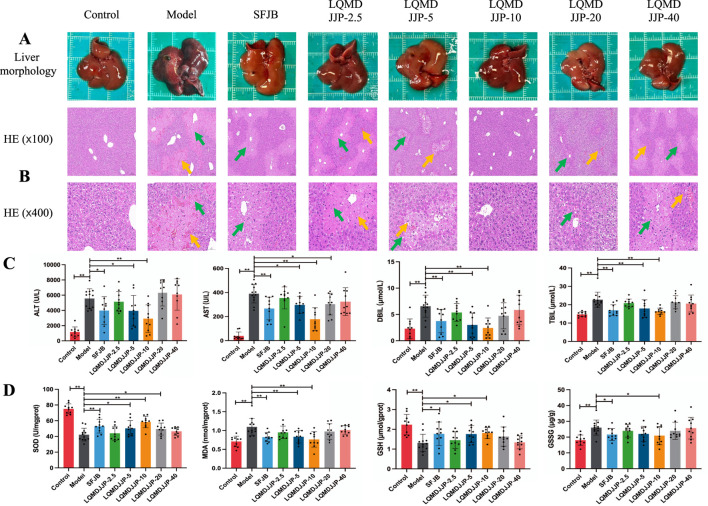
Pathological changes and changes of each index in C57BL/6 mice with acute liver injury induced by APAP. **(A)** Liver morphology of C57BL/6 mice in each group, **(B)** HE pathological staining of C57BL/6 mice in each group under 100 times and 400 times microscopic observation. **(C)** Changes of serum biochemical indexes of C57BL/6 mice in each group. **(D)** Changes in liver oxidative stress indicators in each group of C57BL/6 mice. The green arrows represent liver cell necrosis, the yellow arrows represent hepatic sinusoids and central venous congestion. Only the model group had a sample size of 12, while the other groups had a sample size of 10. Compared with the model group, *means *P* < 0.05, **means *P* < 0.01.

**FIGURE 4:**
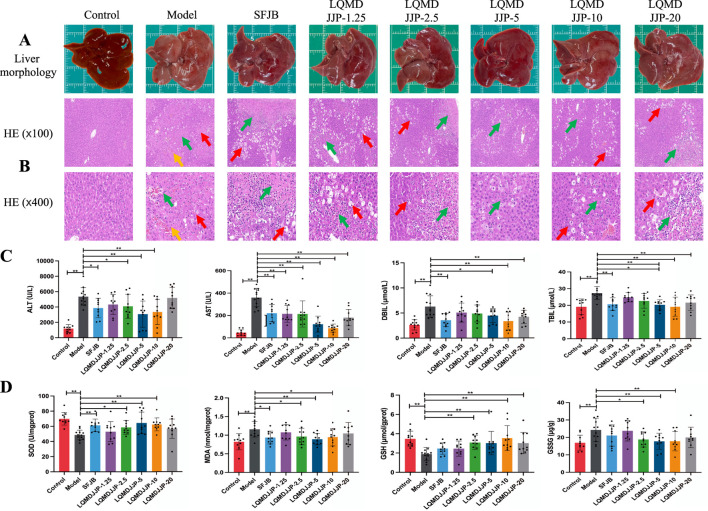
Pathological changes and changes of each index in rats with acute liver injury induced by CCl_4_. **(A)** Liver morphology of SD rats in each group. **(B)** HE pathological staining of SD rats in each group under 100 times and 400 times microscopic observation. **(C)** Changes of serum biochemical indexes of SD rats in each group. **(D)** Changes of oxidative stress indexes in liver of SD rats in each group. The green arrows represent liver cell necrosis, the yellow arrows represent hepatic sinusoids and central venous congestion, and the red arrows represent liver cell degeneration. Only the model group had a sample size of 12, while the other groups had a sample size of 10. Compared with the model group, *means *P* < 0.05, **means *P* < 0.01.

The liver morphology of C57BL/6 mice with APAP-induced acute liver injury is shown in Figure 3A, while the liver morphology of SD rats with CCl_4_-induced acute liver injury is presented in Figure 4A. Observing the liver morphology and liver organ index, it is evident that different doses of LQMDJJP can enhance the liver morphology in both SD rats and C57BL/6 mice.

In the APAP-induced acute liver injury model in C57BL/6 mice, both the SFJB group and all doses of LQMDJJP were effective in reducing the increase in liver index caused by APAP administration. The effect of the LQMDJJP-10 group was similar to that of the SFJB group, with the LQMDJJP-10 group showing the most significant effect.

In the CCl_4_-induced acute liver injury model in SD rats, the SFJB group, as well as the LQMDJJP-2.5, LQMDJJP-5, LQMDJJP-10, and LQMDJJP-20 groups, effectively reduced the liver index increase caused by CCl_4_ intraperitoneal injection. The effect of the LQMDJJP-10 group was similar to that of the SFJB group.

### 3.3 Pathological protective effect of LQMDJJP on acute liver injury

The HE pathological score data for APAP-induced acute liver injury in mice are presented in Table 3 and Figure 2B, with liver pathology images shown in Figure 3B. The HE pathological score data for CCl_4_-induced acute liver injury in rats are provided in Table 4 and Figure 2C, with liver pathology images in Figure 4B. Pathological analysis indicated that LQMDJJP exhibited a degree of protective effect on the liver in both mice and rats across different doses. In the model group, significant liver damage was observed, with hepatocyte necrosis zones around the central vein or near the capsule. The morphological structure of hepatocytes in the necrotic areas appeared blurred, nuclei were dissolved or disappeared, the cytoplasm was eosinophilic, and local inflammatory cell infiltration, as well as bleeding and congestion, was noted. LQMDJJP effectively improved these lesions and reduced the HE pathological scores. In the APAP-induced acute liver injury model in mice, the LQMDJJP-10 group showed the best results, while in the CCl_4_-induced acute liver injury model in rats, the LQMDJJP-5 group exhibited the most effective results.

**TABLE 3:** Dose-effect relationship of LQMDJJP in the treatment of APAP-induced liver injury in C57BL/6 mice. HE pathological score, serum biochemical indexes and liver oxidative stress indexes in C57BL/6 mice (n = 10–12).

Group	Dose (mg/kg/d)	HE scores	ALT	AST	TBIL	DBIL	SOD	MDA	GSH	GSSG
Control	——	0.041 ± 0.003^**^	1166.84 ± 659.61^**^	39.24 ± 32.60^**^	14.76 ± 1.32^**^	2.31 ± 1.83^**^	75.11 ± 6.32^**^	0.71 ± 0.14^**^	2.23 ± 0.52^**^	18.09 ± 3.24^**^
Model	——	0.061 ± 0.002	5559.52 ± 1276.81	390.23 ± 77.11	22.75 ± 4.12	6.57 ± 2.09	42.30 ± 8.13	1.10 ± 0.23	1.31 ± 0.44	26.03 ± 4.73
SFJB	70.00	0.046 ± 0.003^**^	3975.08 ± 1829.13^*^	267.29 ± 94.26^**^	16.99 ± 3.00^**^	3.74 ± 2.22^**^	53.16 ± 8.76^**^	0.83 ± 0.12^**^	1.77 ± 0.60^*^	21.63 ± 3.79^*^
LQMDJJP-2.5	83.33	0.056 ± 0.005^*^	5145.41 ± 1327.33	354.82 ± 91.57	20.87 ± 2.20	5.37 ± 1.68	44.07 ± 8.84	0.95 ± 0.17	1.46 ± 0.42	23.97 ± 4.01
LQMDJJP-5	166.66	0.050 ± 0.003^**^	3947.98 ± 2012.89^*^	297.10 ± 70.03^*^	17.94 ± 4.73^**^	3.01 ± 2.27^**^	50.39 ± 9.72^*^	0.83 ± 0.16^**^	1.76 ± 0.43^*^	22.02 ± 4.78
LQMDJJP-10	333.33	0.045 ± 0.005^**^	2922.86 ± 1802.97^**^	178.19 ± 95.66^**^	16.44 ± 1.73^**^	2.43 ± 1.90^**^	58.34 ± 7.33^**^	0.76 ± 0.21^**^	1.82 ± 0.27^*^	20.93 ± 5.79^*^
LQMDJJP-20	666.66	0.053 ± 0.003^**^	6309.14 ± 1742.38	303.97 ± 90.31^*^	21.37 ± 3.54	4.74 ± 2.48	49.44 ± 7.64^*^	0.98 ± 0.21	1.63 ± 0.48	23.96 ± 5.42
LQMDJJP-40	1333.32	0.057 ± 0.004^*^	6083.34 ± 2060.27	324.50 ± 115.32	20.66 ± 4.62	5.85 ± 2.81	46.51 ± 5.13	1.02 ± 0.11	1.34 ± 0.33	25.67 ± 6.89

Note: Compared with the model group, *means *P* < 0.05, **means *P* < 0.01. Only the model group had a sample size of 12, while the other groups had a sample size of 10. HE, score is based on the degree of sinusoidal congestion in hepatocytes, vacuolar degeneration of hepatocytes, and hepatocyte necrosis. Each parameter is scored on a scale from 0 (none) to 4 (severe), with scores assigned according to the degree of manifestation. The final Suzuki score is the sum of the individual parameter scores, used to quantify the overall severity of liver damage.

**TABLE 4:** Dose-effect relationship of LQMDJJP in the treatment of CCl_4_-induced liver injury in rats HE pathological score, serum biochemical indexes and liver oxidative stress indexes of rats in the study (n = 10–12).

Group	Dose (mg/kg/d)	HE scores	ALT	AST	TBIL	DBIL	SOD	MDA	GSH	GSSG
Control	——	0.025 ± 0.002^**^	1195.70 ± 519.38^**^	47.06 ± 26.19^**^	19.16 ± 4.68^**^	2.64 ± 1.05^**^	69.99 ± 8.38^**^	0.82 ± 0.22^**^	3.47 ± 0.79^**^	16.95 ± 4.29^**^
Model	——	0.043 ± 0.004	5351.33 ± 1174.88	360.17 ± 82.54	27.35 ± 3.86	6.29 ± 2.15	48.61 ± 6.37	1.16 ± 0.21	1.90 ± 0.68	24.32 ± 6.73
SFJB	35.00	0.033 ± 0.003^**^	3872.08 ± 1256.09^*^	220.86 ± 76.53^**^	20.64 ± 3.89^**^	3.58 ± 1.31^**^	61.31 ± 8.30^**^	0.94 ± 0.17^*^	2.46 ± 0.59	21.13 ± 6.12
LQMDJJP-1.25	41.66	0.041 ± 0.003	4330.97 ± 1303.97	214.71 ± 75.77^**^	24.79 ± 2.73	5.09 ± 1.90	52.97 ± 13.17	1.08 ± 0.21	2.46 ± 0.79	23.78 ± 5.83
LQMDJJP-2.5	83.33	0.039 ± 0.002^*^	4092.17 ± 1566.51^*^	213.80 ± 116.86^**^	22.66 ± 4.45^*^	4.91 ± 1.69	58.62 ± 9.09^*^	0.96 ± 0.22^*^	3.09 ± 0.74^**^	18.82 ± 4.34^*^
LQMDJJP-5	166.65	0.037 ± 0.006^**^	3071.66 ± 1704.53^**^	122.21 ± 71.66^**^	20.30 ± 3.04^**^	4.48 ± 1.39^*^	64.47 ± 14.29^**^	0.89 ± 0.15^**^	3.03 ± 1.21^**^	17.77 ± 4.60^**^
LQMDJJP-10	333.30	0.033 ± 0.005^**^	3350.64 ± 1650.18^**^	85.98 ± 35.61^**^	19.13 ± 5.76^**^	3.38 ± 1.80^**^	62.47 ± 8.54^**^	0.95 ± 0.24^*^	3.54 ± 1.28^**^	17.87 ± 5.58^**^
LQMDJJP-20	666.60	0.038 ± 0.004^*^	5176.14 ± 1388.24	180.44 ± 73.47^**^	21.55 ± 4.65^**^	4.22 ± 1.30^**^	56.80 ± 12.78	1.04 ± 0.29	3.03 ± 1.11^**^	20.04 ± 5.89

Note: Compared with the model group, *means *P* < 0.05, **means *P* < 0.01. Only the model group had a sample size of 12, while the other groups had a sample size of 10. HE, score is based on the degree of sinusoidal congestion in hepatocytes, vacuolar degeneration of hepatocytes, and hepatocyte necrosis. Each parameter is scored on a scale from 0 (none) to 4 (severe), with scores assigned according to the degree of manifestation. The final Suzuki score is the sum of the individual parameter scores, used to quantify the overall severity of liver damage.

### 3.4 Changes of serum biochemical indexes

The serum liver function indicators are presented in Tables 3, 4, with the statistical analysis results shown in Figures 3C, 4C.

In the APAP-induced acute liver injury model in C57BL/6 mice, the silibinin group, LQMDJJP-5 group, and LQMDJJP-10 group effectively reduced serum ALT levels, with the LQMDJJP-10 group showing the most significant effect. The SFJB group, LQMDJJP-5 group, LQMDJJP-10 group, and LQMDJJP-20 group significantly reduced serum AST levels, with the LQMDJJP-10 group demonstrating the best results. Additionally, the SFJB group, LQMDJJP-5 group, and LQMDJJP-10 group were effective in lowering serum TBIL levels, with the LQMDJJP-10 group showing the best effect. Similarly, the SFJB group, LQMDJJP-5 group, and LQMDJJP-10 group effectively reduced serum DBIL levels, with the LQMDJJP-10 group providing the best results.

In the CCl_4_-induced acute liver injury model in SD rats, the SFJB group, LQMDJJP-2.5 group, LQMDJJP-5 group, and LQMDJJP-10 group effectively lowered ALT levels in the serum of SD rats, with the LQMDJJP-5 group showing the most pronounced effect. The SFJB group, LQMDJJP-1.25 group, LQMDJJP-2.5 group, LQMDJJP-5 group, LQMDJJP-10 group, and LQMDJJP-20 group significantly reduced serum AST levels, with the LQMDJJP-10 group and LQMDJJP-5 group showing similar effectiveness. The SFJB group, LQMDJJP-2.5 group, LQMDJJP-5 group, LQMDJJP-10 group, and LQMDJJP-20 group effectively reduced serum TBIL levels, with the LQMDJJP-10 group and LQMDJJP-5 group exhibiting comparable results. Similarly, the SFJB group, LQMDJJP-5 group, LQMDJJP-10 group, and LQMDJJP-20 group effectively reduced serum DBIL levels, with the LQMDJJP-10 group and LQMDJJP-5 group showing identical effects.

### 3.5 Changes of liver oxidative stress index

The liver oxidative stress indicators are presented in Tables 3, 4, with the results displayed in Figures 3D, 4D.

In the APAP-induced acute liver injury model in C57BL/6 mice, the SFJB group, LQMDJJP-5 group, LQMDJJP-10 group, and LQMDJJP-20 group effectively increased SOD levels in liver tissue, with the LQMDJJP-10 group showing the most significant effect. The SFJB group, LQMDJJP-5 group, and LQMDJJP-10 group effectively reduced MDA levels in liver tissue, with the LQMDJJP-10 group being the most effective. Additionally, the SFJB group, LQMDJJP-5 group, and LQMDJJP-10 group effectively increased GSH levels in liver tissue, with the LQMDJJP-10 group showing the best results. Both the SFJB group and LQMDJJP-10 group effectively decreased GSSG levels in liver tissue, with the LQMDJJP-10 group showing the best outcome.

In the CCl_4_-induced acute liver injury model in SD rats, the SFJB group, LQMDJJP-2.5 group, LQMDJJP-5 group, and LQMDJJP-10 group effectively increased SOD levels in SD rat serum, with the LQMDJJP-5 group showing the most significant effect. The SFJB group, LQMDJJP-2.5 group, LQMDJJP-5 group, and LQMDJJP-10 group effectively reduced MDA levels in the serum of SD rats, with the LQMDJJP-5 group demonstrating the best results. The LQMDJJP-2.5 group, LQMDJJP-5 group, LQMDJJP-10 group, and LQMDJJP-20 group effectively increased GSH levels in SD rat liver tissue, with the LQMDJJP-10 and LQMDJJP-5 groups showing similar effects. The LQMDJJP-2.5 group, LQMDJJP-5 group, and LQMDJJP-10 group effectively reduced GSSG levels in rat liver tissue, with the LQMDJJP-5 group showing the best result.

### 3.6 Summary of dose-effect relationship

**FIGURE 5:**
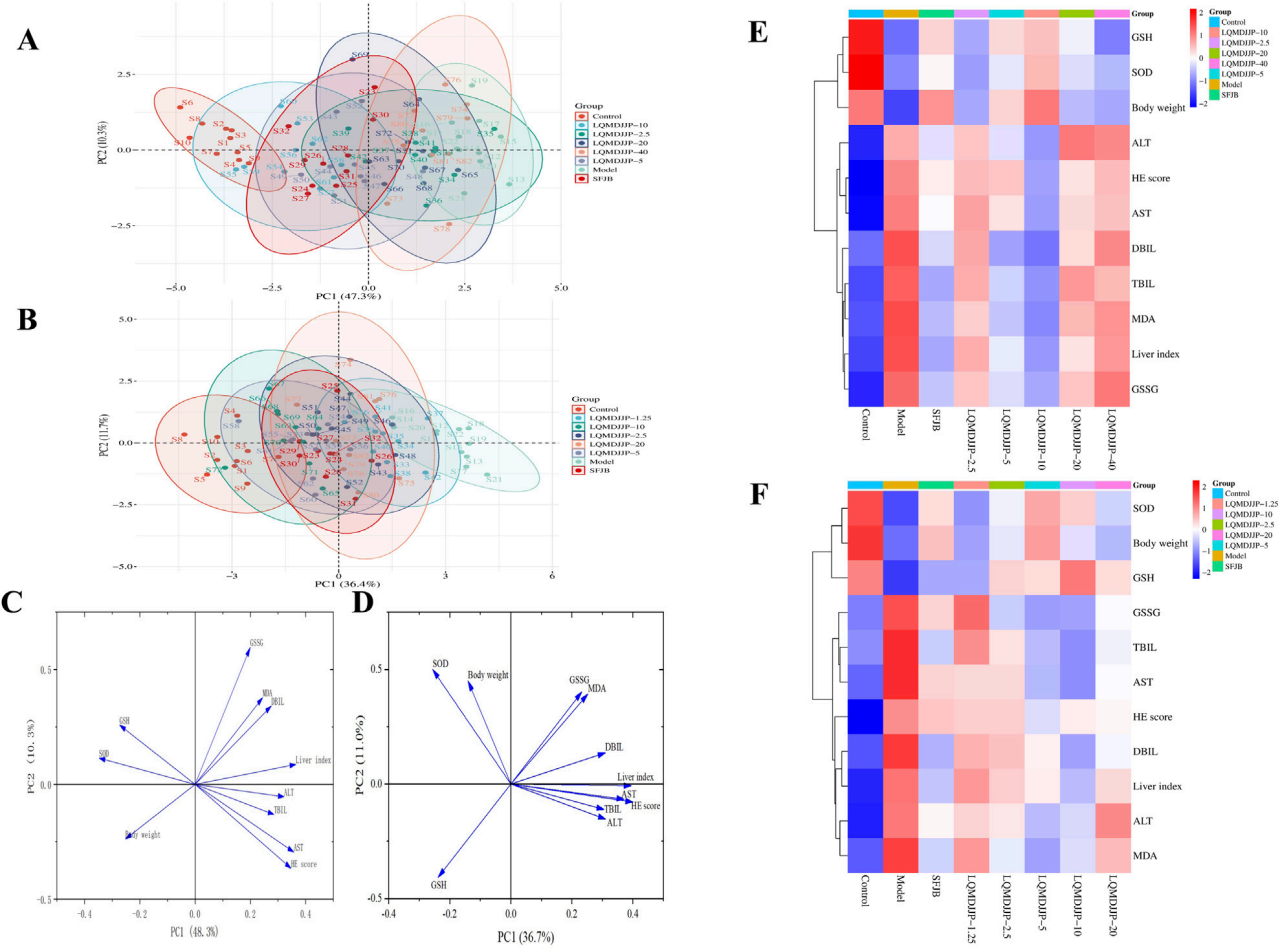
Summary of dose-effect relationship. **(A)** PCA analysis of APAP-induced acute liver injury in C57BL/6 mice. **(B)** PCA analysis of CCl_4_-induced acute liver injury in SD rats. **(C)** The load diagram of each index in C57BL/6 mice with acute liver injury induced by APAP. **(D)** The load diagram of each index in SD rats with acute liver injury induced by CCl_4_. **(E)** Heat map analysis of each index in C57BL/6 mice with acute liver injury induced by APAP. **(F)** Heat map analysis of each index in SD rats with acute liver injury induced by CCl_4_.

PCA and heat map analyses were conducted on 11 indicators from both models. The PCA score plot, loading plot, and heatmap analysis for APAP-induced acute liver injury in C57BL/6 mice are presented in Figures 5A,C,E. The PCA score, loading plot, and heat map analysis for CCl_4_-induced acute liver injury in SD rats are displayed in Figures 5B,D,F.

In APAP-induced acute liver injury in C57BL/6 mice, a comprehensive analysis of 11 measurement indicators in eight groups showed that there was no intersection between the confidence ellipse of the control group and the confidence ellipse of the model group, so the liver injury of the model group was obvious. There was no intersection between the confidence ellipse of the model group and the LQMDJJP-10 group, indicating that the LQMDJJP-10 group did have a good effect on APAP-induced liver injury in C57BL/6 mice. The confidence ellipse of the model group was intersected with the SFJB group, the LQMDJJP-2.5 group, the LQMDJJP-5 group, the LQMDJJP-20 group and the LQMDJJP-40 group. However, the confidence ellipse intersection area with the model group was from large to small: the LQMDJJP-2.5 group, the LQMDJJP-40 group, the LQMDJJP-20 group, the LQMDJJP-5 group, and the SFJB group. The therapeutic effect on liver injury is inversely proportional to the intersection area. In summary, the PCA-X principal component analysis method was used to comprehensively analyze the 11 measurement indexes of eight groups. The results showed that the efficacy intensity of the treatment of liver injury in C57BL/6 mice was from strong to weak: the LQMDJJP-10 group, the SFJB group, the LQMDJJP-5 group, the LQMDJJP-20 group, the LQMDJJP-40 group and the LQMDJJP-2.5 group. The load diagram shows that the distance from the point 0 of the coordinate diagram from far to near point variables are liver index, AST, HE scores, SOD, ALT, GSH, weight, TBIL, DBIL, MDA, GSSG. The results showed that the index of ‘liver index’ had the greatest contribution rate and played an important role in the evaluation of the treatment of APAP-induced liver injury in mice. The results of heat map showed that the body weight of mice was positively correlated with the content of SOD and GSH. The liver index, ALT, AST, TBIL and DBIL in serum, MDA and GSSG in liver tissue and HE score were negatively correlated with the content of SOD and GSH in liver tissue. The reason for this result may be that when APAP was used to replicate the acute liver injury model, various pathological changes such as oxidative stress and inflammation occurred in the body, resulting in reduced food intake, decreased body weight and body temperature, and increased liver index in mice. When given LQMDJP, it can appropriately alleviate the weight loss caused by modeling in mice. SOD and GSH are substances that have a protective effect on the liver. MDA and GSSG are pathogenic or harmful metabolites of the liver. After administration of LQMDJP, the levels of SOD and GSH in the body can be restored, the excess MDA and GSSG in the body can be eliminated, and the liver index of mice can be reduced.

In CCl_4_-induced acute liver injury in rats, the 11 measurement indexes of eight groups were comprehensively analyzed. It was found that there was no intersection between the confidence ellipse of the control group and the confidence ellipse of the model control group, which indicated that the liver injury of the model control group was obvious. The confidence ellipse of the model group was intersected with the SFJB group and each dose group of LQMDJJP. However, the confidence ellipse intersection area with the model group was from large to small: the LQMDJJP-1.25 group, the LQMDJJP-2.5 group, the LQMDJJP-20 group, the SFJB group, the LQMDJJP-10 group and the LQMDJJP-5 group. The therapeutic effect of each group of drugs on liver injury in rats was compared with the intersection area. In summary, the PCA-X principal component analysis method was used to comprehensively analyze the 11 measurement indicators of eight groups. The results showed that the efficacy intensity of the treatment of liver injury in rats was from strong to weak: the LQMDJJP-5 group, the LQMDJJP-10 group, the SFJB group, the LQMDJJP-20 group, the LQMDJJP-2.5 group, and the LQMDJJP-1.25 group. The load diagram showed that the variables from far to near from the 0 point of the coordinate diagram were HE score, liver index, AST, ALT, DBIL, TBIL, MDA, SOD, GSH, GSSG and body weight. The results showed that the index of ‘HE scores’ had the greatest contribution rate and played an important role in the evaluation of the treatment of CCl_4_-induced liver injury in rats. The results of heat map showed that the body weight of rats was positively correlated with the content of SOD and GSH. The liver index, ALT, AST, TBIL, DBIL, MDA and GSSG in serum were negatively correlated with the content of SOD and GSH in liver tissue. The reason for this result is similar to that of using APAP to replicate the acute liver injury model. After LQMDJJP, the body’s protective substances for the liver are increased, the content of substances that are harmful to the liver is reduced, and the body weight and liver index of rats are improved.

### 3.7 Results of non-targeted metabolomics

**FIGURE 6:**
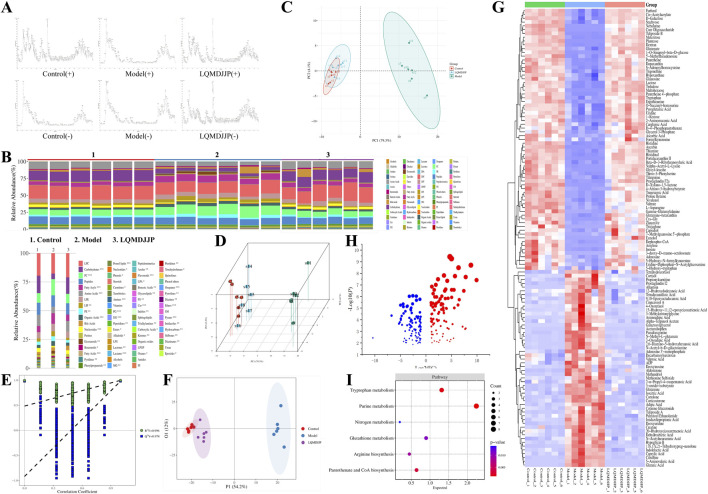
Results of non-targeted metabolomics study. **(A)** TIC diagram of mice in each group. **(B)** Component analysis of metabolites in each group and each sample. **(C,D)** PCA score plots of 2D and 3D. **(E,F)** OPLS-DA 2D score plot. **(G)** Heat map analysis of differential metabolites. **(H)** The bubble diagram of differential metabolites. **(I)** KEGG analysis and bubble diagram of different metabolic pathways.

The liver tissue of mice was analyzed using UPLC-Q-TOF-MS metabolomics. The TIC chromatogram of liver tissue samples from each group is presented in Figure 6A. The relative abundance of various metabolites in each group of samples, as well as the relative abundance of different metabolite types in each sample, are shown in Figure 6B. The HMDB database was used to identify the chromatographic peaks through mass spectrometry analysis, and the metabolic components in the liver tissue of LQMDJJP-10 treated mice after modeling and administration were identified. The relative percentage content was calculated using peak area normalization. Figures 6C,D display the 2D PCA score plot, 3D PCA score plot, and box plot of the corresponding principal component scores, respectively. The results indicated that the PCA scores of the liver tissues from the APAP intraperitoneal injection model group and the animals treated with LQMDJJP-10 after APAP injection were distinctly separated, suggesting that the metabolites between the groups exhibited significant differences. The results of the OPLS-DA permutation test are presented in Figure 6E. The OPLS-DA 2D score plot is shown in Figure 6F, and these results align with the PCA findings. Significant differences in liver tissue metabolomics data were observed between the APAP intraperitoneal injection model group and the animals treated with LQMDJJP-10 after APAP injection, suggesting that LQMDJJP-10 can effectively alter liver metabolism and mitigate acute liver injury.

**FIGURE 7:**
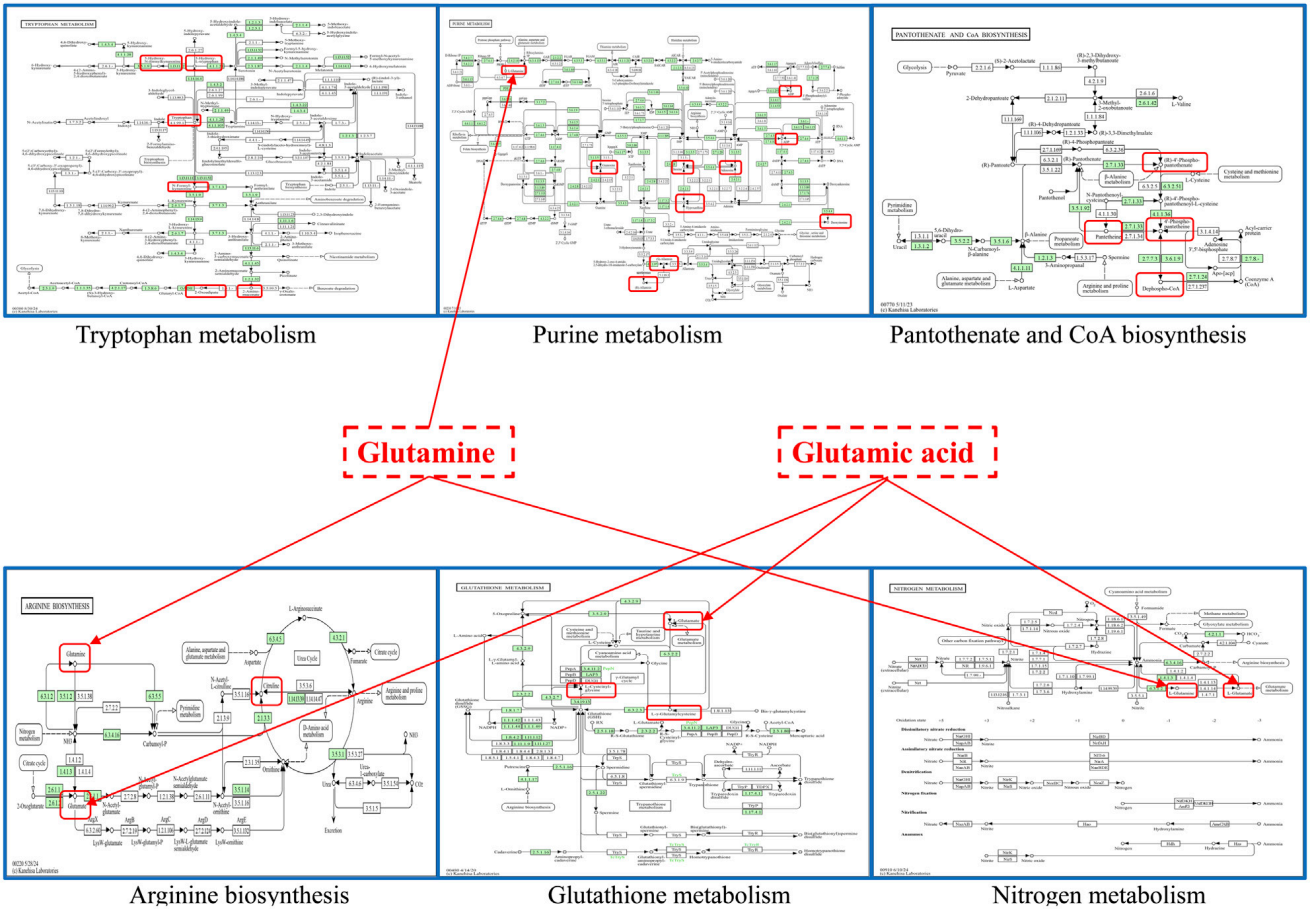
The obtained six important metabolic pathways and metabolic differential metabolites were obtained. Metabolism pathway map from KEGG website, URL: https://www.kegg.jp/kegg/.

A total of 849 compounds were detected, and a comparison was made between the metabolites of the model group and the LQMDJJP-10 group after treatment. Differential metabolites of liver tissue were selected based on the criteria of *P* < 0.05, FC ≥ 2 or ≤ 0.5, and VIP > 1, with corresponding HMDB and KEGG numbers. A total of 121 liver differential metabolites were identified, as summarized in Table 5. The heat map of differential metabolites is presented in Figure 6G, while the volcano plot of differential metabolites is shown in Figure 6H. When compared to the model group, the LQMDJJP-10 group exhibited an increase in metabolites located in the upper right corner, while metabolites in the upper left corner decreased. A total of 121 differential metabolites were input into the Metabo Analyst 5.0 platform for metabolic pathway analysis. This analysis identified six differential metabolic pathways, as summarized in Table 6 and illustrated in Figures 6I, 7.

**TABLE 5:** 121 differential metabolites that met the screening conditions.

No.	Metabolite	HMDB	KEGG	Control	Model	LQMDJJP	Model/Control			LQMDJJP/Model		
							FC	VIP	Trend	FC	VIP	Trend
1	Trigonelline	HMDB0000875	C01004	2.3261 ± 0.3049	1.1265 ± 0.2542	2.2989 ± 0.2103	0.4843	1.2633	↓^**^	2.0407	1.3509	↑^**^
2	Pseudoecgonine	HMDB0006348	C12449	0.0053 ± 0.0052	0.1608 ± 0.0516	0.0078 ± 0.0061	51.6083	1.2266	↑^**^	0.0492	1.2855	↓^**^
3	Prostaglandin F2α	HMDB0014440	C01516	8.4165 ± 0.5346	0.6414 ± 0.2140	7.7679 ± 2.8801	0.0762	1.3521	↓^**^	12.1108	1.2859	↑^**^
4	Palmitoyl Ethanolamide	HMDB0002100	C16512	0.4553 ± 0.0488	0.7914 ± 0.1323	0.3870 ± 0.0676	1.8018	1.2176	↑^**^	0.4925	1.3075	↓^**^
5	Histidinal	HMDB0012234	C01929	0.1441 ± 0.0130	0.0107 ± 0.0072	0.1380 ± 0.0253	0.0743	1.3472	↓^**^	13.6439	1.3805	↑^**^
6	2-Aminomuconic Acid	HMDB0001241	C02220	0.2066 ± 0.0540	0.0336 ± 0.0232	0.2312 ± 0.0426	0.1627	1.2494	↓^**^	6.8781	1.3677	↑^**^
7	Tranexamic Acid	HMDB0014447	C12535	3.9413 ± 0.5414	0.4473 ± 0.1591	2.6839 ± 0.7187	0.1135	1.3362	↓^**^	5.9998	1.3257	↑^**^
8	Histidine	HMDB0000177	C00135	9.7888 ± 0.9887	1.6099 ± 0.4920	8.2951 ± 0.9669	0.1645	1.3415	↓^**^	5.1526	1.4016	↑^**^
9	Pantetheine	HMDB0003426	C00831	1.2922 ± 0.1661	0.2546 ± 0.0857	1.0617 ± 0.1998	0.1971	1.33	↓^**^	4.1693	1.3457	↑^**^
10	Tilarginine	HMDB0029416	C03884	0.2822 ± 0.0349	0.0636 ± 0.0247	0.2359 ± 0.0429	0.2255	1.3217	↓^**^	3.7076	1.3465	↑^**^
11	Glutamine-betaxanthin	HMDB0304684	C08568	2.7537 ± 0.2394	1.0111 ± 0.1319	2.5893 ± 0.6038	0.3672	1.3349	↓^**^	2.5609	1.2447	↑^**^
12	Tryptophan	HMDB0000929	C00078	5.9347 ± 0.6317	2.4181 ± 0.5162	5.5902 ± 0.9567	0.4284	1.3109	↓^**^	2.3118	1.334	↑^**^
13	Creatine	HMDB0000064	C00300	11.9754 ± 7.5121	31.8080 ± 8.0568	8.9762 ± 2.0938	3.1584	1.1169	↑^**^	0.2822	1.3014	↓^**^
14	Citrulline	HMDB0000904	C00327	0.0173 ± 0.0093	0.4410 ± 0.1151	0.0200 ± 0.0098	25.5331	1.2896	↑^**^	0.0454	1.3618	↓^**^
15	5-Hydroxy-tryptophan	HMDB0000472	C00643	0.0667 ± 0.0311	0.0295 ± 0.0230	0.0675 ± 0.0179	0.4426	0.8286	↓^*^	2.2871	1.013	↑^**^
16	4-Amino-3-hydroxybutyrate	HMDB0061877	C03678	0.0693 ± 0.0147	0.0045 ± 0.0036	0.0464 ± 0.0238	0.0868	1.307	↓^**^	8.8262	1.1639	↑^**^
17	12-Hydroxydodecanoic Acid	HMDB0002059	C08317	0.2398 ± 0.0480	0.4626 ± 0.1940	0.1402 ± 0.0859	1.9294	0.8603	↑^*^	0.303	1.0673	↓^**^
18	Portulacaxanthin II	HMDB0012281	C08565	0.4515 ± 0.0688	0.0042 ± 0.0059	0.3917 ± 0.0965	0.0045	1.3321	↓^**^	198.1251	1.3577	↑^**^
19	Pantetheine 4′-phosphate	HMDB0001416	C01134	0.3464 ± 0.0621	0.0177 ± 0.0240	0.3608 ± 0.0795	0.0134	1.3234	↓^**^	75.7079	1.3599	↑^**^
20	Dopaxanthin	HMDB0012221	C08543	0.1817 ± 0.0381	0.0101 ± 0.0141	0.1664 ± 0.0453	0.013	1.3044	↓^**^	72.5751	1.3166	↑^**^
21	Nalpha-Acetyl-L-Lysine	HMDB0000446	C12989	0.4120 ± 0.1117	0.0103 ± 0.0078	0.3411 ± 0.0664	0.0194	1.2887	↓^**^	42.2887	1.3891	↑^**^
22	Ergothioneine	HMDB0003045	C05570	5.8464 ± 1.4215	0.4836 ± 0.3828	5.0448 ± 1.2991	0.0749	1.2787	↓^**^	11.2296	1.3304	↑^**^
23	Proline Betaine	HMDB0004827	C10172	1.1998 ± 0.2011	0.1593 ± 0.0554	0.8623 ± 0.2333	0.1405	1.3204	↓^**^	5.0479	1.3191	↑^**^
24	Glutamate	HMDB0000148	C00025	59.7269 ± 2.9591	23.8159 ± 6.2132	63.5012 ± 6.0811	0.4085	1.3199	↓^**^	2.7698	1.3703	↑^**^
25	O-Succinyl-homoserine	HMDB0255868	C01118	1.4089 ± 0.0674	0.7202 ± 0.2124	1.4445 ± 0.1796	0.4499	1.271	↓^**^	2.2269	1.3074	↑^**^
26	3-Methylcrotonylglycine	HMDB0000459	C20828	0.4642 ± 0.0323	0.8699 ± 0.3506	0.4068 ± 0.0560	1.7168	0.9082	↑^**^	0.4934	1.0385	↓^**^
27	Aminoadipic Acid	HMDB0000510	C00956	0.3523 ± 0.0117	1.4240 ± 0.5631	0.3437 ± 0.0273	3.5807	1.1298	↑^**^	0.2707	1.208	↓^**^
28	Methionine Sulfoxide	HMDB0002005	C02989	1.0092 ± 0.3199	3.8516 ± 1.7421	0.9300 ± 0.2841	3.5024	1.0576	↑^**^	0.2447	1.121	↓^**^
29	N-Methyl-L-glutamate	HMDB0062660	C01046	3.9325 ± 1.1164	55.1680 ± 16.6836	3.4021 ± 1.0930	15.5788	1.24	↑^**^	0.0528	1.3106	↓^**^
30	Glutamine	HMDB0000641	C00064	0.2512 ± 0.2032	2.3796 ± 0.2956	0.3240 ± 0.5387	9.4736	1.3299	↑^**^	0.05	1.3303	↓^**^
31	2,6-Diamino-5-hydroxyhexanoic Acid	HMDB0000450	C16741	0.1286 ± 0.0709	2.8677 ± 1.2168	0.0736 ± 0.0580	25.9192	1.1725	↑^**^	0.0236	1.2525	↓^**^
32	5-Aminovaleric Acid	HMDB0003355	C00431	0.0022 ± 0.0020	0.3266 ± 0.1156	0.0031 ± 0.0019	143.6996	1.236	↑^**^	0.0113	1.3085	↓^**^
33	Carglumic Acid	HMDB0015673	C05829	0.1666 ± 0.0238	0.0635 ± 0.0311	0.1657 ± 0.0513	0.3814	1.2422	↓^**^	2.6078	1.1677	↑^**^
34	D-4′-Phosphopantothenate	HMDB0001016	C03492	4.9147 ± 0.9386	2.1206 ± 0.7130	5.6494 ± 1.4014	0.4315	1.1999	↓^**^	2.664	1.25	↑^**^
35	L-Asparagine	HMDB0000168	C00152	0.1754 ± 0.0189	0.0475 ± 0.0091	0.1092 ± 0.0242	0.2705	1.3307	↓^**^	2.302	1.2502	↑^**^
36	Pyroglutamic Acid	HMDB0000267	C01879	1.3577 ± 0.0589	0.7178 ± 0.2261	1.4397 ± 0.1922	0.4851	1.2474	↓^**^	2.0058	1.2873	↑^**^
37	Allantoin	HMDB0000462	C01551	0.3102 ± 0.0407	9.8636 ± 3.4207	0.3653 ± 0.1060	31.4163	1.2587	↑^**^	0.0395	1.3234	↓^**^
38	Formylkynurenine	HMDB0060485	C02700	0.0902 ± 0.0366	0.0170 ± 0.0235	0.0974 ± 0.0561	0.068	1.0904	↓^**^	14.8715	1.0264	↑^**^
39	Stachyose	HMDB0003553	C01613	52.1602 ± 7.3806	12.4354 ± 7.1858	47.6040 ± 6.4250	0.2384	1.2754	↓^**^	3.8281	1.3441	↑^**^
40	Planteose	HMDB0302793	C03848	1.6368 ± 0.2794	0.0033 ± 0.0048	1.4768 ± 0.1792	0.0008	1.3163	↓^**^	1068.0827	1.4014	↑^**^
41	Dextran	HMDB0247628	C00372	78.9369 ± 5.2101	0.3248 ± 0.6093	72.1961 ± 11.1215	0.0011	1.3501	↓^**^	867.6098	1.3974	↑^**^
42	Trehalose	HMDB0000975	C01083	2.0405 ± 0.2387	0.1260 ± 0.2172	2.2791 ± 0.2885	0.0062	1.3278	↓^**^	190.0948	1.4032	↑^**^
43	Amylose	HMDB0003403	C00718	0.2924 ± 0.1029	0.0021 ± 0.0031	0.1629 ± 0.0450	0.0029	1.2369	↓^**^	175.2676	1.3403	↑^**^
44	3-deoxy-D-manno-octulosonate	HMDB0304125	C01187	7.6981 ± 4.0705	0.0215 ± 0.0201	4.7797 ± 2.3426	0.0034	1.1254	↓^**^	173.762	1.1877	↑^**^
45	Melezitose	HMDB0011730	C08243	86.5402 ± 11.4562	2.7154 ± 4.6826	87.6254 ± 7.7421	0.0059	1.3289	↓^**^	165.3352	1.4107	↑^**^
46	Maltohexaose	HMDB0012253	C01936	1.8940 ± 0.3041	0.0425 ± 0.0619	2.2016 ± 0.3227	0.0097	1.3269	↓^**^	129.4082	1.3968	↑^**^
47	Lactose	HMDB0000186	C00243	42.2042 ± 2.7232	3.0062 ± 3.9255	44.0325 ± 7.2112	0.03	1.3461	↓^**^	36.7044	1.3872	↑^**^
48	1-Kestose	HMDB0011729	C03661	34.4866 ± 4.5119	4.5553 ± 6.9025	35.8983 ± 5.4082	0.0424	1.2839	↓^**^	25.4193	1.3505	↑^**^
49	Tuliposide B	HMDB0303141	C08570	0.9135 ± 0.1138	0.0666 ± 0.0066	0.7748 ± 0.1061	0.0732	1.3366	↓^**^	11.3915	1.398	↑^**^
50	Core Oligosaccharide	HMDB0013471	c0338	4.0755 ± 0.6166	0.2666 ± 0.0264	3.0611 ± 0.9391	0.0691	1.3216	↓^**^	10.6846	1.3177	↑^**^
51	Glycerol 3-Phosphate	HMDB0000126	C00093	1.0983 ± 0.3859	0.2077 ± 0.1075	1.2018 ± 0.3829	0.1642	1.1827	↓^**^	6.4663	1.2563	↑^**^
52	D-Galactose	HMDB0250761	C00124	43.3729 ± 1.5163	20.0253 ± 5.0192	40.2949 ± 2.0104	0.4591	1.3175	↓^**^	2.0201	1.3701	↑^**^
53	N-Acetyl-b-D-galactosamine	HMDB0000853	C05021	0.2618 ± 0.0303	1.8490 ± 0.6201	0.2471 ± 0.1151	7.0612	1.2075	↑^**^	0.1253	1.2782	↓^**^
54	Cotinine Glucuronide	HMDB0001013	C00190	0.1931 ± 0.1050	1.4204 ± 0.7496	0.1790 ± 0.0811	6.1518	1.0802	↑^**^	0.1238	1.1567	↓^**^
55	Tuliposide A	HMDB0303140	C08561	0.0037 ± 0.0022	0.1665 ± 0.0738	0.0176 ± 0.0217	35.8007	1.1913	↑^**^	0.062	1.2195	↓^**^
56	5-Hydroxy-N-formylkynurenine	HMDB0004086	C05648	1.2446 ± 0.4631	0.5506 ± 0.1297	1.2093 ± 0.3614	0.4424	1.0219	↓^**^	2.1962	1.1059	↑^**^
57	N-Acetylneuraminic Acid	HMDB0000230	C00270	0.2651 ± 0.0527	0.6093 ± 0.1225	0.2840 ± 0.0909	2.298	1.2339	↑^**^	0.4661	1.2488	↓^**^
58	Valproic Acid	HMDB0000901	C07185	0.0291 ± 0.0156	0.0657 ± 0.0176	0.0257 ± 0.0069	2.2551	1.0295	↑^**^	0.3907	1.214	↓^**^
59	7-Methylguanosine 5′-phosphate	HMDB0059612	C03998	0.1640 ± 0.0595	0.0064 ± 0.0119	0.1637 ± 0.1151	0.0039	1.2158	↓^**^	232.8191	1.0227	↑^**^
60	Zanamivir	HMDB0014698	C08095	3.8524 ± 1.1101	0.6478 ± 0.1827	2.5791 ± 0.9856	0.1681	1.2379	↓^**^	3.7363	1.1758	↓^**^
61	Propionylcarnitine	HMDB0000824	C03017	0.4447 ± 0.0857	1.1946 ± 0.4462	0.5401 ± 0.1275	2.6865	1.1026	↑^**^	0.4521	1.0932	↓^**^
62	Thiamine	HMDB0000235	C00378	0.7186 ± 0.0654	0.1075 ± 0.0413	0.6079 ± 0.1238	0.1495	1.347	↓^**^	5.2472	1.3678	↑^**^
63	Tetradecanedioic Acid	HMDB0000872	C11002	0.6163 ± 0.1242	1.4638 ± 0.6238	0.5521 ± 0.2911	2.3752	0.9546	↑^**^	0.3772	1.0049	↓^**^
64	Adipic Acid	HMDB0000448	C06104	4.4816 ± 1.4750	27.2815 ± 10.3810	5.5434 ± 3.5521	6.0875	1.1914	↑^**^	0.1955	1.2094	↓^**^
65	20-Hydroxyeicosatetraenoic Acid	HMDB0005998	C14748	1.3685 ± 0.8487	4.6108 ± 2.0339	0.7421 ± 0.2912	3.3692	1.0278	↑^**^	0.1427	1.2096	↓^**^
66	9,10-Epoxyoctadecanoic Acid	HMDB0061650	C19418	2.0965 ± 0.3062	5.5446 ± 1.5892	2.1568 ± 1.1613	2.6447	1.1496	↑^**^	0.389	1.1362	↓^**^
67	Caprylic Acid	HMDB0000482	C06423	0.0237 ± 0.0069	0.0842 ± 0.0263	0.0216 ± 0.0036	3.5458	1.189	↑^**^	0.2562	1.2817	↓^**^
68	Prostaglandin I2	HMDB0001335	C01312	0.0683 ± 0.0278	0.7476 ± 0.2571	0.1184 ± 0.0779	10.951	1.2425	↑^**^	0.1584	1.2616	↓^**^
69	Trisjuglone	HMDB0030568	C10187	0.7423 ± 0.3463	0.0308 ± 0.0031	0.5689 ± 0.2626	0.0416	1.1259	↓^**^	16.7883	1.169	↑^**^
70	Imidazolepropionic Acid	HMDB0002271	C20522	0.0791 ± 0.0157	0.3516 ± 0.0784	0.0576 ± 0.0179	4.7526	1.2733	↑^**^	0.1492	1.3447	↓^**^
71	Indolelactic Acid	HMDB0000671	C02043	0.1913 ± 0.0402	0.5631 ± 0.1337	0.1584 ± 0.0642	2.9441	1.227	↑^**^	0.2814	1.3048	↓^**^
72	D-Xylono-1,5-lactone	HMDB0011676	C02266	0.9496 ± 0.1471	0.0128 ± 0.0113	0.6035 ± 0.1766	0.0136	1.3273	↓^**^	47.2933	1.3407	↑^**^
73	S-Adenosylhomocysteine	HMDB0000939	C00021	7.5070 ± 1.1923	2.1819 ± 0.5284	6.9533 ± 1.4047	0.2907	1.305	↓^**^	3.1868	1.3348	↑^**^
74	Galactosylglycerol	HMDB0006790	C05401	0.0135 ± 0.0203	8.4969 ± 5.5310	0.0063 ± 0.0044	2268.6595	1.0251	↑^**^	7.00E-04	1.0882	↓^**^
75	Inosine	HMDB0000195	C00294	127.6631 ± 36.6192	12.1413 ± 5.1247	100.2871 ± 23.4593	0.1113	1.2596	↓^**^	8.26	1.3373	↑^**^
76	Uridine	HMDB0000296	C00299	4.0450 ± 0.5114	1.9724 ± 0.5763	4.2577 ± 0.5384	0.4876	1.2348	↓^**^	2.1587	1.3221	↑^**^
77	Deoxyinosine	HMDB0000071	C05512	0.0567 ± 0.0065	0.2124 ± 0.0962	0.0573 ± 0.0163	4.025	1.0227	↑^*^	0.2233	1.0629	↓^**^
78	Adenosine	HMDB0000050	C00212	2.8871 ± 0.9219	1.1782 ± 0.2550	2.5710 ± 0.7092	0.4157	1.1142	↓^**^	2.2053	1.1859	↑^**^
79	Nebularine	HMDB0029956	C01736	6.6000 ± 0.5099	0.0238 ± 0.0431	4.0859 ± 0.8382	0.0007	1.346	↓^**^	811.817	1.3799	↑^**^
80	Dephospho-CoA	HMDB0001373	C00882	10.3349 ± 3.0180	1.8453 ± 0.3722	7.8643 ± 1.3664	0.1959	1.2438	↓^**^	4.2945	1.3566	↑^**^
81	Uridine-Diphosphate-N-Acetylglucosamine	HMDB0000290	C00043	3.0321 ± 1.2107	0.8404 ± 0.3150	2.6192 ± 1.2432	0.257	1.1024	↓^**^	2.9509	1.0167	↑^**^
82	Deoxyuridine	HMDB0000012	C00526	0.1197 ± 0.0396	1.1543 ± 0.3090	0.1449 ± 0.0402	9.3026	1.2816	↑^**^	0.1368	1.3436	↓^**^
83	Guanosine	HMDB0000133	C00387	1.2806 ± 0.2459	0.5379 ± 0.2070	1.2008 ± 0.1729	0.4201	1.2074	↓^**^	2.2323	1.2798	↑^**^
84	5′-Methylthioadenosine	HMDB0001173	C00170	0.7997 ± 0.1376	0.2065 ± 0.0544	0.6904 ± 0.0999	0.2582	1.2822	↓^**^	3.3439	1.3519	↑^**^
85	ADP	HMDB0001341	C00008	0.4147 ± 0.1636	1.1472 ± 0.3993	0.4846 ± 0.1751	2.842	1.0448	↑^**^	0.4136	1.0349	↓^**^
86	Adenosine 3′-monophosphate	HMDB0003540	C01367	9.5897 ± 2.0041	22.3828 ± 5.3122	10.7593 ± 3.2743	2.334	1.1608	↑^**^	0.3812	1.1437	↓^**^
87	Isocitric Acid	HMDB0001874	C00311	88.3451 ± 13.2274	205.4940 ± 15.4768	90.8795 ± 23.7782	2.326	1.314	↑^**^	0.4422	1.3496	↓^**^
88	Glutaric Acid	HMDB0000661	C00489	1.2315 ± 0.1106	9.1651 ± 2.5113	1.1978 ± 0.0715	7.4423	1.2565	↑^**^	0.1307	1.3331	↓^**^
89	Cis-Acetylacrylate	HMDB0060461	C07091	0.8618 ± 0.0591	0.3642 ± 0.0807	0.8172 ± 0.0288	0.4197	1.3186	↓^**^	2.2164	1.3992	↑^**^
90	15-Hydroxy-11,12-epoxyeicosatrienoic Acid	HMDB0005050	C14781	0.6382 ± 0.1129	6.4447 ± 3.8175	0.6784 ± 0.3702	10.2378	1.0368	↑^**^	0.1232	1.1072	↓^**^
91	2-Oxoadipic Acid	HMDB0000225	C00322	0.2873 ± 0.0294	2.5913 ± 1.1028	0.3045 ± 0.0708	9.0733	1.1431	↑^**^	0.1177	1.2096	↓^**^
92	3-ureido-isobutyrate	HMDB0304156	C05100	0.0573 ± 0.0513	1.1779 ± 0.2588	0.0897 ± 0.1814	25.5434	1.2889	↑^**^	0.0182	1.3137	↓^**^
93	2-n-Propyl-4-oxopentanoic Acid	HMDB0060683	C16655	0.0290 ± 0.0097	0.0684 ± 0.0131	0.0294 ± 0.0090	2.355	1.2059	↑^**^	0.4299	1.2692	↓^**^
94	Furfural	HMDB0032914	C14279	0.4795 ± 0.0407	0.1459 ± 0.0551	0.4516 ± 0.0281	0.3043	1.3171	↓^**^	3.0943	1.3932	↑^**^
95	Threo-3-Phenylserine	HMDB0002184	C03290	0.1823 ± 0.0422	0.0118 ± 0.0107	0.1645 ± 0.0356	0.0505	1.3004	↓^**^	13.952	1.3659	↑^**^
96	Glycyl-leucine	HMDB0000759	C02155	0.4481 ± 0.1336	0.0112 ± 0.0102	0.3725 ± 0.0852	0.018	1.2754	↓^**^	43.9389	1.3728	↑^**^
97	Hypoglycin B	HMDB0029428	C08280	0.0273 ± 0.0094	0.1695 ± 0.0667	0.0180 ± 0.0087	6.3188	1.1804	↑^**^	0.0906	1.2712	↓^**^
98	Cys-Gly	HMDB0000078	C01419	2.3353 ± 0.5158	0.0556 ± 0.0559	2.0713 ± 0.8866	0.0132	1.3146	↓^**^	37.2729	1.2069	↑^**^
99	Gamma-Glutamylalanine	HMDB0006248	C03740	0.4065 ± 0.0945	0.0473 ± 0.0146	0.2347 ± 0.0749	0.1311	1.272	↓^**^	4.9606	1.2572	↑^**^
100	Esmolol	HMDB0014333	C06980	0.0416 ± 0.0106	0.0025 ± 0.0002	0.0549 ± 0.0262	0.0613	1.2937	↓^**^	22.4247	1.2007	↑^**^
101	Acetaminophen	HMDB0001859	C06804	0.1185 ± 0.0267	3.2090 ± 1.2287	0.1070 ± 0.0107	28.3418	1.2111	↑^**^	0.0319	1.2754	↓^**^
102	Decarbamoylsaxitoxin	HMDB0038319	C20021	0.0280 ± 0.0234	0.0686 ± 0.0146	0.0229 ± 0.0093	2.4509	1.0239	↑^**^	0.3348	1.2885	↓^**^
103	1-O-Sinapoyl-beta-D-glucose	HMDB0302379	C01175	7.5461 ± 1.1321	0.7437 ± 0.0738	7.3943 ± 1.8301	0.0948	1.3207	↓^**^	9.7165	1.3465	↑^**^
104	Nivalenol	HMDB0004304	C06080	0.3016 ± 0.0222	0.0291 ± 0.0252	0.2354 ± 0.0588	0.0965	1.3434	↓^**^	8.0837	1.3268	↑^**^
105	Valtrate	HMDB0034493	C09801	0.1474 ± 0.0178	0.0258 ± 0.0121	0.0883 ± 0.0129	0.1749	1.328	↓^**^	3.4237	1.3401	↑^**^
106	Aucubin	HMDB0036562	C09771	1.8221 ± 0.1664	0.0168 ± 0.0221	1.4107 ± 0.1772	0.0043	1.3441	↓^**^	183.036	1.4028	↑^**^
107	Alpha-Terpineol Acetate	HMDB0032051	C12300	0.3905 ± 0.0810	0.8382 ± 0.2094	0.3632 ± 0.0567	2.1445	1.1488	↑^**^	0.4778	1.2512	↓^**^
108	Dehydroabietic Acid	HMDB0061925	C12078	0.5922 ± 0.2860	2.8669 ± 1.1273	0.3656 ± 0.1847	5.1978	1.1339	↑^**^	0.1044	1.2435	↓^**^
109	4-Oxoretinol	HMDB0012329	C16683	0.1905 ± 0.0656	2.5341 ± 1.8030	0.1630 ± 0.0393	11.3768	0.9773	↑^**^	0.0719	1.0632	↓^**^
110	Capsidiol	HMDB0002352	C09627	0.0611 ± 0.0084	0.0168 ± 0.0071	0.0749 ± 0.0326	0.2742	1.2926	↓^**^	4.4721	1.1285	↑^**^
111	Cinncassiol A	HMDB0035164	C17645	0.8853 ± 0.0909	1.8294 ± 0.4047	0.8120 ± 0.2194	2.0664	1.1722	↑^**^	0.4439	1.2315	↓^**^
112	Beta-D-3-Ribofuranosyluric Acid	HMDB0029920	C05513	0.6073 ± 0.1076	0.1375 ± 0.0444	0.5291 ± 0.0558	0.2264	1.3002	↓^**^	3.8481	1.3894	↑^**^
113	Hypoxanthine	HMDB0000157	C00262	46.3380 ± 2.3039	20.8014 ± 8.8515	43.4129 ± 5.7866	0.4774	1.2522	↓^**^	2.087	1.2393	↑^**^
114	Corticosterone	HMDB0001547	C02140	0.1212 ± 0.0891	0.9429 ± 0.1901	0.0992 ± 0.0470	7.7827	1.2859	↑^**^	0.1052	1.3635	↓^**^
115	Tetrahydrocortisol	HMDB0000949	C05472	0.1853 ± 0.0595	0.4877 ± 0.0637	0.2420 ± 0.1510	2.7425	1.289	↑^**^	0.4957	1.0771	↓^**^
116	Aldosterone	HMDB0000037	C01780	0.0255 ± 0.0110	0.3585 ± 0.1654	0.0924 ± 0.0925	18.1941	1.1095	↑^**^	0.2579	1.0054	↓^**^
117	Cortisol	HMDB0000063	C00735	0.0161 ± 0.0075	0.2924 ± 0.2072	0.0205 ± 0.0182	13.2157	0.9908	↑^**^	0.0756	1.0074	↓^**^
118	Cortolone	HMDB0003128	C05481	0.0612 ± 0.0327	0.8021 ± 0.2075	0.0651 ± 0.0413	13.1092	1.2686	↑^**^	0.0631	1.3355	↓^**^
119	Methandriol	HMDB0254517	C14493	0.0151 ± 0.0131	0.5142 ± 0.2211	0.0201 ± 0.0153	43.5969	1.1467	↑^**^	0.0391	1.1917	↓^**^
120	11b,17a,21-Trihydroxypreg-nenolone	HMDB0006760	C05489	0.0070 ± 0.0041	0.6516 ± 0.1773	0.0207 ± 0.0112	104.1054	1.2893	↑^**^	0.0285	1.3547	↓^**^
121	Ascorbic Acid	HMDB0000044	C00072	0.7427 ± 0.1681	0.1169 ± 0.0400	0.5138 ± 0.2898	0.1574	1.2847	↓^**^	4.3957	1.0472	↑^**^

Note: Because APAP-induced acute liver injury is very common in real life, and our subsequent mechanism research is also centered on the model of APAP-induced acute liver injury, we only studied the model of APAP-induced acute liver injury in metabolomics research. Only one LQMDJJP administration group was selected for the study. The basis for selecting this group was based on the previous pharmacodynamic study. We found that this group (LQMDJJP 10 times clinical dose group) was the best dose group for the treatment of APAP-induced acute liver injury in mice in five LQMDJJP dose groups.

**TABLE 6:** six metabolic pathways with p value less than 0.05.

No.	Metabolic pathways	Participating metabolites	Total	Hits	Expected	Raw *p*	-Log(*p*)
1	Purine metabolism	Glutamine; ADP; Adenosine; Deoxyinosine; Hypoxanthine; Inosine; Guanosine; Allantoin	70	8	2.2425	0.0013	2.8856
2	Tryptophan metabolism	L-Tryptophan; 5-Hydroxy-L-tryptophan; L-Formylkynurenine; Oxoadipic Acid; 5-Hydroxy-N-formylkynurenine; 2-Aminomuconic Acid	41	6	1.3134	0.0015	2.8160
3	Pantothenate and CoA biosynthesis	Dephospho-CoA; Pantetheine 4′-phosphate; Pantetheine; D-4′-Phosphopantothenate	20	4	0.6407	0.0031	2.5087
4	Arginine biosynthesis	Glutamate; Citrulline; Glutamine	14	3	0.44849	0.0088	2.0561
5	Glutathione metabolism	Glutamic Acid; Cysteinylglycine; 5-Oxoproline; Gamma-Glutamylalanine	28	4	0.89698	0.0108	1.9651
6	Nitrogen metabolism	Glutamic Acid; Glutamine	6	2	0.19221	0.0139	1.8569

Glutathione, as an anti-oxidative stress substance, is composed of glutamic acid, gysteine and glycine, and is consumed in large quantities during acute liver injury (Lan et al., 2025; Wang et al., 2022). Glutamine can be converted into glutamic acid by deamination in mitochondria, which is one of the precursors for the synthesis of glutathione (Ling et al., 2023). Among the 121 differential metabolites and six differential metabolic pathways that meet the conditions, the differential metabolites that are mainly involved in glutathione metabolism and alleviate liver oxidative stress include glutamic acid, glutamine, gamma-glutamylalanine, etc. The differential metabolic pathways involved in alleviating liver oxidative stress are purine metabolism, tryptophan metabolism, pantothenate and CoA biosynthesis, arginine biosynthesis, glutathione metabolism and nitrogen metabolism.

Uric acid produced by purine metabolism directly scavenges ROS at physiological concentration and reduces free radical generation by regulating xanthine oxidase activity (Boardman et al., 2020). Tryptophan metabolism activates SIRT1 by converting to NAD^+^, enhances the expression of antioxidant enzymes such as SOD and cat, and balances the Pro/antioxidant effect of kynurenine pathway. Pantothenic acid and C_O_A biosynthesis maintain fatty acid oxidation and TCA cycle homeostasis, prevent lipotoxic ROS and support glutathione synthesis (Slyshenkov et al., 2004). Arginine metabolism regulates vascular function by detoxifying ammonia and generating nitric oxide through the urea cycle, and induces polyamines to activate autophagy and clear damaged mitochondria (Zhang et al., 2024). Glutathione metabolism, as a core antioxidant system, directly scavenges ROS and relies on NADPH regeneration system to maintain redox balance. Its key enzymes GCLC and GCLM are regulated by Nrf2. Nitrogen metabolism reduces ammonia accumulation and avoids mitochondrial oxidative damage through urea cycle and glutamine synthesis (Abiko et al., 2023). These pathways are interrelated (for example, NAD^+^ enhances the SIRT1/Nrf2 axis to promote glutathione synthesis, and C_O_A supports fatty acid metabolism to reduce lipid peroxidation), so as to jointly build a multi-level antioxidant network.

According to the results of pathology, serum biochemical markers, liver antioxidant indicators, and non-targeted metabolomics, the results demonstrated that LQMDJJP could mitigate acute liver injury and was categorized under anti-oxidative stress mechanism. Consequently, the Keap1-Nrf2 antioxidant protection pathway was selected for further investigation.

### 3.8 Effect of LQMDJJP on the mRNA expression of anti-oxidative stress targets related to Keap1-Nrf2 signaling pathway

**FIGURE 8:**
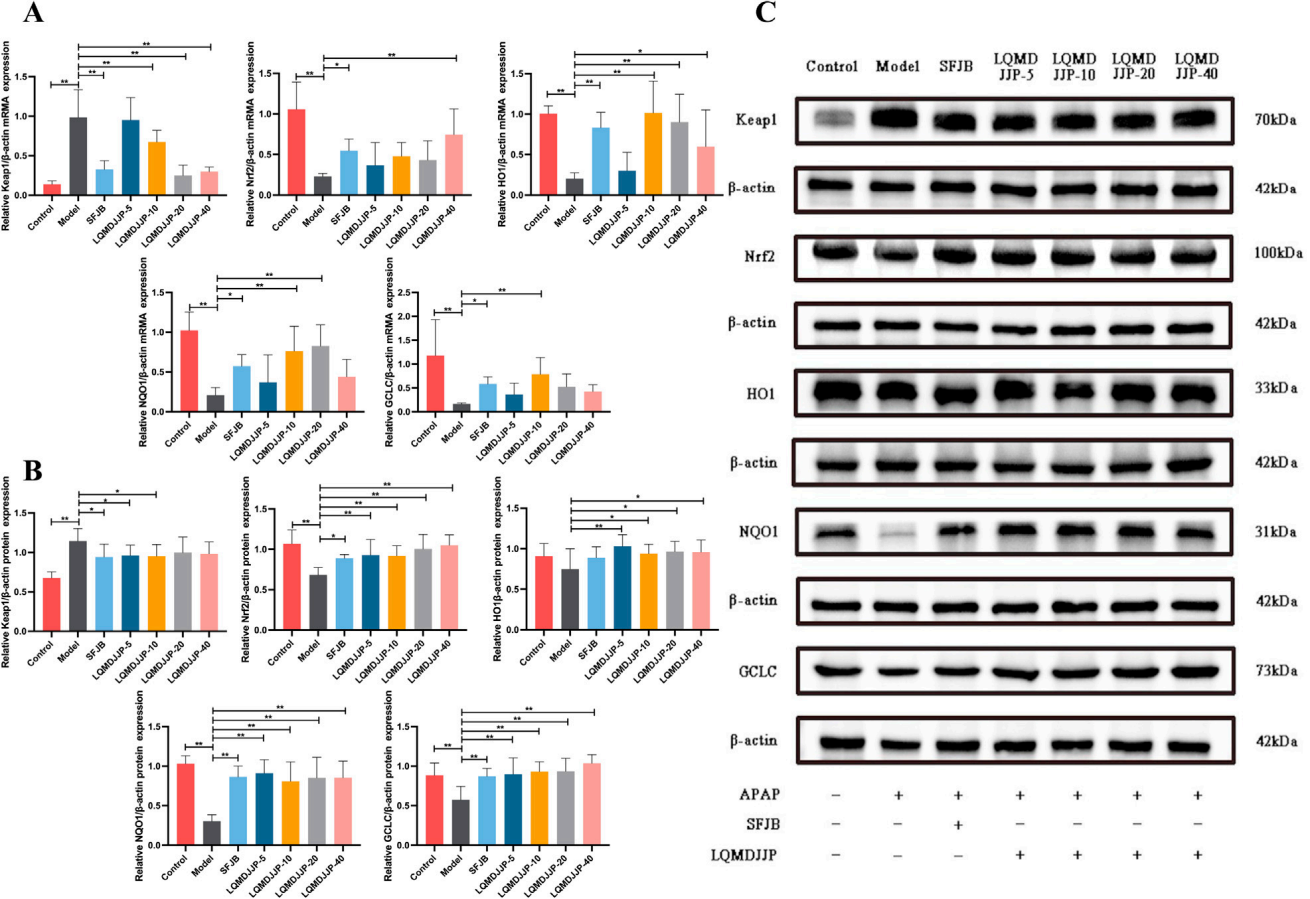
The impact of LQMDJJP on gene expressions of mRNA levels and protein expression in the liver. **(A)** The mRNA expression of antioxidant stress factors Keap1, Nrf2, HO1, NQOI, and GCLC in the liver. **(B–C)** The protein expression of antioxidant stress factors Keap1, Nrf2, HO1, NQOI, and GCLC in the liver. Compared with the model group, *means *P* < 0.05, **means *P* < 0.01.

The PCR results of oxidative stress-related mRNA are shown in Figure 8A and Table 7. In APAP-induced acute liver injury model in mice, compared with the control group, the relative expression of Keap1 mRNA in the model group increased significantly (*P* < 0.01), and the relative expression of Nrf2, HO1, NQO1 and GCLC mRNA in the model group decreased significantly (*P* < 0.01). Compared with the model group, the relative expression of Keap1 mRNA in the SFJB group, the LQMDJJP-5 group, the LQMDJJP-10 group, the LQMDJJP-20 group and the LQMDJJP-40 group decreased significantly (*P* < 0.01). Compared with the model group, the relative expression of Nrf2 mRNA in the LQMDJJP-40 group was significantly increased (*P* < 0.01), and the relative expression of Nrf2 mRNA in the SFJB group was increased (*P* < 0.05). Compared with the model control group, the relative expression of HO1 mRNA in the SFJB group, the LQMDJJP-10 group and the LQMDJJP-20 group increased significantly (*P* < 0.01), and the relative expression of Nrf2 mRNA in the 40-fold dose group increased (*P* < 0.05). Compared with the model control group, the relative expression of NQO1 mRNA in the LQMDJJP-10 group and the LQMDJJP-20 group increased significantly (*P* < 0.01), and the relative expression of NQO1 mRNA in the SFJB group increased (*P* < 0.05). Compared with the model control group, the relative expression of GCLC mRNA in the LQMDJJP-10 group increased significantly (*P* < 0.01), and the relative expression of GCLC mRNA in the SFJB group increased (*P* < 0.05).

**TABLE 7:** The mRNA expression of antioxidant stress factors Keap1, Nrf2, HO1, NQOI, and GCLC in the liver in mice (n = 6).

Group	Dose (mg/kg/d)	Keap1	Nrf2	HO1	NQO1	GCLC
Control	——	0.14 ± 0.04**	1.06 ± 0.34**	1.00 ± 0.10**	1.02 ± 0.23**	1.18 ± 0.75**
Model	——	0.98 ± 0.35	0.23 ± 0.04	0.20 ± 0.07	0.21 ± 0.09	0.16 ± 0.02
SFJB	70.00	0.33 ± 0.11**	0.55 ± 0.14*	0.83 ± 0.19**	0.57 ± 0.14*	0.58 ± 0.15*
LQMDJJP-5	166.66	0.95 ± 0.28	0.37 ± 0.28	0.30 ± 0.23	0.37 ± 0.35	0.36 ± 0.23
LQMDJJP-10	333.33	0.67 ± 0.15**	0.48 ± 0.17	1.02 ± 0.39**	0.76 ± 0.31**	0.79 ± 0.35**
LQMDJJP-20	666.66	0.25 ± 0.13**	0.43 ± 0.24	0.90 ± 0.34**	0.83 ± 0.27**	0.52 ± 0.27
LQMDJJP-40	1333.32	0.30 ± 0.06**	0.75 ± 0.32**	0.60 ± 0.45*	0.44 ± 0.22	0.42 ± 0.14

Note: Compared with the model group, *means *P* < 0.05, **means *P* < 0.01.

### 3.9 Effect of LQMDJJP on the protein expression of anti-oxidative stress target related to Keap1-Nrf2 signaling pathway

The WB results of oxidative stress-related proteins are shown in Figures 8B,C and Table 8. In APAP-induced acute liver injury model in mice, compared with the control group, the relative expression of Keap1 protein in the model group increased significantly (*P* < 0.01), and the relative expression of Nrf2, NQO1 and GCLC protein in the model group decreased significantly (*P* < 0.01). The relative expression of HO1 protein in the model group only showed a downward trend (*P* > 0.05). Compared with the model group, the relative expression of Keap1 protein in the SFJB group, the LQMDJJP-5 group and the LQMDJJP-10 group decreased (*P* < 0.05). Compared with the model group, the relative expression of Nrf2 protein in the LQMDJJP-5 group, the LQMDJJP-10 group, the LQMDJJP-20 group and the LQMDJJP-40 group decreased significantly (*P* < 0.01), and the relative expression of Nrf2 protein in the SFJB group decreased (*P* < 0.05). Compared with the model group, the relative expression of HO1 protein in the LQMDJJP-5 group decreased significantly (*P* < 0.01), and the relative expression of HO1 protein in the LQMDJJP-10 group, the LQMDJJP-20 group and the LQMDJJP-40 group decreased (*P* < 0.05). Compared with the model group, the relative expression levels of NQO1 and GCLC proteins in the SFJB group, the LQMDJJP-5 group the LQMDJJP-10 group, the LQMDJJP-20 group and the LQMDJJP-40 group decreased significantly (*P* < 0.01).

**TABLE 8:** The protein expression of antioxidant stress factors Keap1, Nrf2, HO1, NQOI, and GCLC in the liver in mice (n = 6).

Group	Dose (mg/kg/d)	Keap1	Nrf2	HO1	NQO1	GCLC
Control	——	0.68 ± 0.08**	1.07 ± 0.17**	0.91 ± 0.16	1.03 ± 0.10**	0.89 ± 0.15**
Model	——	1.15 ± 0.16	0.68 ± 0.09	0.75 ± 0.25	0.31 ± 0.08	0.57 ± 0.17
SFJB	70.00	0.94 ± 0.16*	0.89 ± 0.05*	0.89 ± 0.13	0.86 ± 0.14**	0.87 ± 0.10**
LQMDJJP-5	166.66	0.96 ± 0.13*	0.93 ± 0.19**	1.03 ± 0.14**	0.91 ± 0.17**	0.90 ± 0.21**
LQMDJJP-10	333.33	0.95 ± 0.15*	0.92 ± 0.13**	0.94 ± 0.12*	0.81 ± 0.24**	0.93 ± 0.12**
LQMDJJP-20	666.66	1.00 ± 0.20	1.01 ± 0.18**	0.96 ± 0.13*	0.85 ± 0.26**	0.94 ± 0.16**
LQMDJJP-40	1333.32	0.98 ± 0.15	1.05 ± 0.13**	0.96 ± 0.15*	0.85 ± 0.21**	1.04 ± 0.11**

Note: Compared with the model group, *means *P* < 0.05, **means *P* < 0.01.

## 4 Discussion

Acute liver injury is a prevalent condition in clinical settings, often resulting from the excessive use of medications or chemical toxicity. Currently, acute liver injury and acute liver failure induced by APAP overdose remain significant challenges in clinical practice (Jaeschke et al., 2020). CCl_4_-induced hepatotoxicity may occur through two main mechanisms. First, CCl_4_ activates cytochrome P_450_ enzymes in liver microsomes, leading to the cleavage of the C-Cl bond in the molecule, which generates chlorine and trichloromethane free radicals. These free radicals can then covalently bind to macromolecules in liver cells and attack unsaturated lipids beneath the plasma membrane, triggering lipid peroxidation (Mohi-Ud-Din et al., 2019). Additionally, CCl_4_ reacts with O_2_ to form a trichloro peroxide free radical, which has strong lipid peroxidation activity *in vivo*. This free radical promotes lipid peroxidation and accelerates cell death. Traditional medicine is widely used in the treatment of liver related diseases and has outstanding effects,and extracts from traditional herbs have also shown promising results.

This article focuses on the therapeutic effects of LQMDJJP on acute liver injury induced by APAP in C57BL/6 mice and acute liver injury induced by CCl_4_ in SD rats, and explores the dose effect relationship of LQMDJJP in treating these two types of acute liver injuries. PCA and thermography were used to analyze whether the clinical dosage was reasonable. Research has found that LQMDJJP can significantly reduce the increase in liver index caused by modeling, significantly reduce weight loss caused by modeling, significantly reduce liver pathological scores and improve liver morphological appearance, significantly reduce liver function indicators such as ALT, AST, DBIL, and TBIL in serum, significantly reduce oxidative stress indicators such as GSSG and MDA in liver tissue, and significantly increase oxidative stress indicators such as GSH and SOD in liver tissue. Through comprehensive analysis of PCA and heatmap, it was found that LQMDJJP showed significant effects in the treatment of APAP induced acute liver injury in mice within the range of 83.33 mg/kg/d-1333.32 mg/kg/d, with the 10 times the clinical dose of LQMDJJP (333.33 mg/kg/d) showing the best effect. LQMDJJP showed significant effects in the treatment of CCl_4_ induced acute liver injury in rats within the range of 41.66 mg/kg/d-666.66 mg/kg/d, with the best effect observed in the 5 times the clinical dose of LQMDJJP (166.66 mg/kg/d). Therefore, the clinical dosage of LQMDJJP (2.00 g/60 kg/d) is reasonable.

The metabolites generated through the tryptophan metabolic pathway, including L-Tryptophan, 5-Hydroxy-L-tryptophan, L-Formylkynurenine, oxoadipic acid, and 5-Hydroxy-N-Formylkynurenine, are crucial in the development of acute liver injury. Alterations in the levels of these metabolites may indicate the activation or suppression of the tryptophan metabolic pathway, thereby influencing liver function. Intestinal flora-derived tryptophan metabolites, such as IAAld and IAA, can activate AhR and facilitate nuclear translocation and Nrf2 activation under normal conditions, thereby helping to mitigate oxidative stress damage and preserve liver homeostasis (Shang et al., 2023). Arginine can promote the production of GSH and protect against oxidative stress by activating the Nrf2 signaling pathway. Supplementing with arginine boosts antioxidant capacity, alleviates oxidative stress, and activates Nrf2, leading to the upregulation of genes and proteins dependent on the antioxidant response element, including GCLC, GS, GR, GST, GPx, CAT, SOD, NQO1, and HO-1 (Liang et al., 2018). GSH is a tripeptide made up of glutamic acid, cysteine, and glycine. The disruption of the balance of GSH precursor amino acids is closely linked to the severity of liver steatosis. In a study by Oren Rom et al., glycine supplementation was shown to regulate fatty acid oxidation and enhance GSH synthesis, thereby improving liver injury (Rom et al., 2020). Clinical studies have demonstrated that orally taking GSH supplements directly does not alleviate oxidative stress (Gyatso, 1991). The metabolism of alanine, aspartate, and glutamate plays a crucial role in oxidative stress and acute liver injury. Alanine metabolism is particularly important for maintaining cellular redox balance. In one study, inhibiting mitochondrial pyruvate carrier and alanine aminotransferase two revealed that the combined deletion of both increased susceptibility to oxidative stress, particularly in an APAP-induced acute liver injury model. This indicates that alanine metabolism is vital for protecting the liver against oxidative damage (Yiew et al., 2023). Histidine metabolism boosts the glutathione antioxidant system, which plays a key role in maintaining the balance between GSH and GSSG. Supplementing with histidine has been shown to increase the activity of antioxidant enzymes such as glutathione transferase and glutathione reductase, helping to preserve the balance of GSH and GSSG and mitigate oxidative stress (Yang et al., 2023).

**FIGURE 9:**
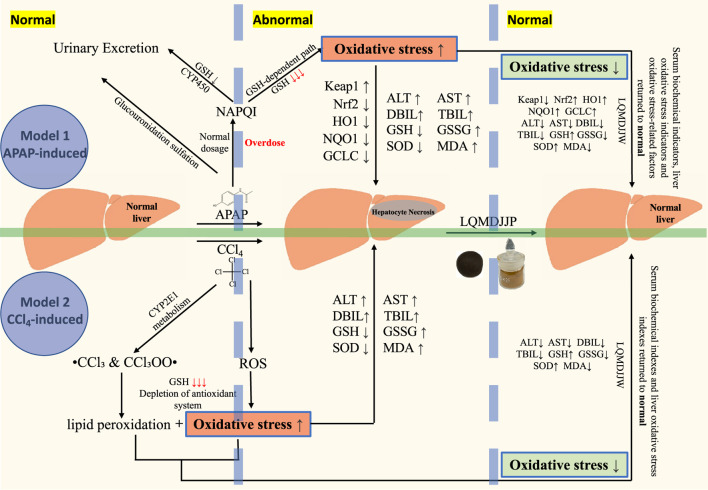
The mechanism of acute liver injury induced by CCl_4_ and APAP, and the mechanism of LQMDJJP in the treatment of acute liver injury by anti-oxidative stress.

In this study, the therapeutic effects of LQMDJJP on liver injury induced by CCl_4_ and APAP were investigated, providing preliminary evidence for its potential scientific application. Metabolomics was used to examine the differences in metabolites before and after treatment, and the findings were validated through molecular biology techniques. The mechanisms underlying acute liver injury caused by CCl_4_ and APAP, as well as the role of LQMDJJP in treating acute liver injury through antioxidative stress, are illustrated in Figure 9. However, a limitation of this study is that APAP-induced liver injury is more commonly encountered in daily life. As a result, this paper focuses solely on the mechanism of liver injury caused by APAP and does not explore liver injury induced by CCl_4_. Additionally, in investigating the mechanism of liver injury, only four dose groups showing better effects were selected from the five dose groups of LQMDJJP for further research. Future research could utilize methods such as UPLC-Q-TOF-MS, GC-MS, ICP-MS, and other analytical techniques to identify and quantify the active components and heavy metals. This approach could help elucidate the relationship between these active components and liver cell repair, as well as investigate their effects on liver cell apoptosis, inflammation, and oxidative stress. It is hoped that these efforts will offer new insights and more effective traditional medical interventions for the treatment of acute liver injury.

The present study demonstrates that Langqing Meiduo Jiujie pills (LQMDJJP) exert protective effects against APAP- and CCl_4_-induced acute liver injury primarily through the Keap1-Nrf2 antioxidant pathway. While the therapeutic effects were not strictly dose-dependent, our multi-dose exploration identified optimal efficacy ranges (e.g., LQMDJJP-10 in mice and LQMDJJP-5 in rats), suggesting a threshold for antioxidant capacity beyond which higher doses may not enhance benefits. Notably, the discordance between mRNA and protein expression of Keap1-Nrf2 components (e.g., Nrf2 protein elevation without mRNA changes in LQMDJJP-5 group) may reflect post-translational regulation via Keap1 degradation or mTOR-mediated translation enhancement, warranting further kinase activity assays. In conclusion, LQMDJJP alleviates acute liver injury by orchestrating multi-pathway antioxidant responses, with the Keap1-Nrf2 axis as a pivotal but non-exclusive mechanism.

## 5 Conclusion

LQMDJJP, a Tibetan medicinal formulation with over 400 years of history, is commonly used to treat hepatobiliary disorders such as hepatitis, acute liver injury, fatty liver, alcoholic liver disease, and liver pain associated with cirrhosis. However, its dose-response relationship and the underlying mechanisms in the treatment of acute liver injury have yet to be documented. This study demonstrated that LQMDJJP has a significant therapeutic effect on acute liver injury induced by CCl_4_ and APAP. The clinical dosage was found to be reasonable, with the recommended daily dose for adults being 2.00 g/60 kg. The results indicated that LQMDJJP could effectively mitigate weight loss associated with liver injury, improve the morphological and histopathological conditions of the liver, significantly reduce serum liver function markers such as ALT, AST, DBIL, and TBIL, and effectively enhance antioxidant levels, such as SOD and GSH, in liver tissue. It can significantly lower the levels of MDA and GSSG in liver tissue, thereby reducing oxidative stress in the liver. Additionally, it has been shown to improve liver metabolism, helping to mitigate acute liver injury caused by drugs. The therapeutic effect on acute liver injury may be linked to the GSH metabolic pathway as well as the metabolism of glutamic acid, glutamine, γ-glutamylalanine, and others. Furthermore, we initially validated its mechanism of antioxidative stress in ameliorating acute liver injury through PCR and Western blot analysis.

## Data Availability

The raw data supporting the conclusions of this article will be made available by the authors, without undue reservation.

## Abbreviations

ALT, Alanine aminotransferase; APAP, Acetaminophen; AST, Aspartate aminotransferase; CCl_4_, Carbon tetrachloride; DBIL, Direct Bilirubin; GSH, Glutathione; GSSG, Oxidized glutathione; GCLC, Glutamate-Cysteine Ligase Catalytic Subunit; H&E, Haematoxylin-eosin; HO-1, Heme Oxygenase 1; Keap1, Kelch-like ECH-associated protein 1; LQMDJJP-X group, X times the clinical dose group of Langqing Meiduo Jiujie pills; MDA, Malondialdehyde; NAPQI, N-acetyl-p-benzoquinone imine; NQO1, NAD(P)H oxidoreductase 1; Nrf2, Nuclear Factor erythroid 2-Related Factor 2; OPLS-DA, orthogonal partial least squares discriminant analysis; PCA, Principal Component Analysis; PZH, Pien Zai Huang; SFJB, silibinin capsules; SOD, Superoxide Dismutase; TBIL, Total Bilirubin; VIP, Variable importance for the projection.
